# Unsupervised Plot-Scale LAI Phenotyping via UAV-Based Imaging, Modelling, and Machine Learning

**DOI:** 10.34133/2022/9768253

**Published:** 2022-07-02

**Authors:** Qiaomin Chen, Bangyou Zheng, Karine Chenu, Pengcheng Hu, Scott C. Chapman

**Affiliations:** ^1^School of Agriculture and Food Sciences, The University of Queensland, St Lucia, QLD, Australia; ^2^Agriculture and Food, CSIRO, Queensland Bioscience Precinct, St Lucia, QLD, Australia; ^3^The University of Queensland, Queensland Alliance for Agriculture and Food Innovation, Toowoomba, QLD, Australia

## Abstract

High-throughput phenotyping has become the frontier to accelerate breeding through linking genetics to crop growth estimation, which requires accurate estimation of leaf area index (LAI). This study developed a hybrid method to train the random forest regression (RFR) models with synthetic datasets generated by a radiative transfer model to estimate LAI from UAV-based multispectral images. The RFR models were evaluated on both (i) subsets from the synthetic datasets and (ii) observed data from two field experiments (i.e., Exp16, Exp19). Given the parameter ranges and soil reflectance are well calibrated in synthetic training data, RFR models can accurately predict LAI from canopy reflectance captured in field conditions, with systematic overestimation for LAI<2 due to background effect, which can be addressed by applying background correction on original reflectance map based on vegetation-background classification. Overall, RFR models achieved accurate LAI prediction from background-corrected reflectance for Exp16 (correlation coefficient (*r*) of 0.95, determination coefficient (*R*^2^) of 0.90~0.91, root mean squared error (RMSE) of 0.36~0.40 m^2^ m^−2^, relative root mean squared error (RRMSE) of 25~28%) and less accurate for Exp19 (*r* =0.80~0.83, *R*^2^ = 0.63~0.69, RMSE of 0.84~0.86 m^2^ m^−2^, RRMSE of 30~31%). Additionally, RFR models correctly captured spatiotemporal variation of observed LAI as well as identified variations for different growing stages and treatments in terms of genotypes and management practices (i.e., planting density, irrigation, and fertilization) for two experiments. The developed hybrid method allows rapid, accurate, nondestructive phenotyping of the dynamics of LAI during vegetative growth to facilitate assessments of growth rate including in breeding program assessments.

## 1. Introduction

Leaf area index (LAI) is defined as one-sided leaf area per unit surface area for field crops with flat leaves [1]. For most applications, LAI refers to the green leaf area contributing to photosynthesis and transpiration, although in remote sensing, the term GAI (Green Area Index) also considers other chlorophyll-containing plant parts such as stems and ears/heads/spikes/flowers [2, 3]. LAI is an important trait for genotype selection and adaptation assessment since it can indicate crop health conditions under biotic and abiotic stresses and contribute to crop growth rate, biomass, and grain yield formulation [4–6]. Precise measurements of LAI can aid in informing the status of the main photosynthesis organs and potential crop growth rate through the season.

Direct measurement of LAI consists of manually harvesting samples in the field, separating leaves off plants, and finally measuring their area with instruments to calculate LAI [7, 8]. Direct measurement is time-consuming, labour-intensive, and expensive for a large number of plots [4]. Being destructive, repeated samples make this method impractical for small breeding plots [9]. In this context, it is necessary to develop faster and more feasible indirect methods for rapid phenotyping as required in modern breeding [10, 11]. Direct measurements are theoretically regarded as more accurate than indirect measurements, given samples are representative to account for the main architecture of the entire canopy [12]. However, an advantage of indirect measurement is that it is possible to measure the entire plot rather than a small sample harvest area. Indirect methods are of two types—under and above the canopy, with the former utilizing approximations and models of light penetration and canopy architecture. However, these under-canopy methods are limited in their ability to sample plots efficiently as they are slow so cannot easily sample entire plots. We focus on above-canopy estimation in this work.

Remote sensing technology has been widely used and become a practical way to retrieve crop LAI from satellite multispectral or hyperspectral data [13–15]. The remote-sensing methods provide a rapid, economic, and nondestructive way to capture crop canopy information at a large scale, which to a large extent has addressed bottlenecks associated with direct measurements [16, 17]. However, currently most satellite data have insufficient temporal and spatial resolution to be utilized in field plot trials. Precise positioning and unmanned aerial vehicle (UAV) technologies facilitate high-throughput phenotyping in agriculture and breeding programs [18–20]. Compared with satellite data, the use of UAV platforms in breeding programs has several advantages. It provides very high spatial resolution data (centimetre to sub-centimetre), which is particularly suitable for small-plot breeding trials [21, 22]. Missions can be scheduled at various heights to balance flight time against ground-scale precision [23]. It is feasible to schedule flights at a required time interval (e.g., daily, weekly) or specific growth stages and to determine the best flight time according to weather (sunny calm conditions) to produce high-quality UAV data [24, 25].

Retrieval methods for extracting crop traits (e.g., LAI) from UAV data are similar to retrieving from satellite data in principles, and their advantages and limitations have been discussed in several reviews [26–29]. Retrieving crop traits from spectral data depends on the established relationship between target trait and raw band reflectance or/and derived vegetation indices (VIs). Based on the source of relationship, retrieval methods can be divided into three categories: (i) empirical method, establishing a relationship based on real experimental data [22, 30]; (ii) physical method, directly using the established cause-effect relationship expressed in radiative transfer models (RTMs) [31–33]; (iii) hybrid method, establishing a relationship from synthetic data generated by RTMs [7, 14, 34]. Compared with empirical methods and physical methods, hybrid methods can balance both general applicability (suitable for application in a wide range of conditions) and computational efficiency [29, 35].

This paper aims to apply a hybrid method to develop a predictive model to rapidly estimate wheat LAI from canopy reflectance collected with proximal (UAV-borne) multispectral cameras (visible to near-infrared range) under varying conditions. To meet breeding needs, the model should be able to distinguish genetic variations and to identify the effects of management treatments on considered genotypes. Here, random forest regression (RFR) models were trained on synthetic datasets generated by a RTM (i.e., PROSAIL model [36]) to predict LAI. These RFR models were then validated against measurements of LAI and UAV-based multispectral data from two trials with different genotypes and management practices in terms of plant density, irrigation, and fertilization. For such a hybrid method, the extent to which the RFR model can accurately predict LAI from experimental data largely depends on the similarity between synthetic multispectral data and observed multispectral data. In response to increasing the data similarity, three potential solutions (i.e., calibration of parameter range, soil reflectance, and image soil background) were implemented to evaluate their effects on improving model prediction accuracy.

## 2. Materials and Methods

### 2.1. Overview

**Figure 1:**
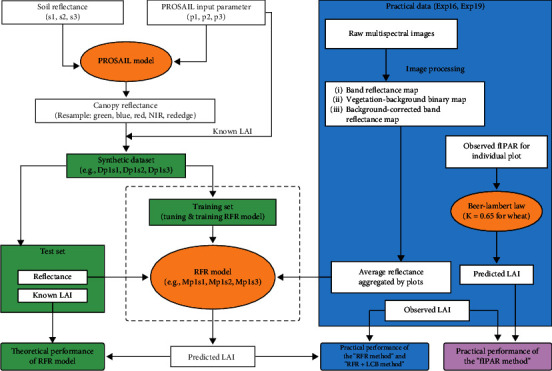
Research flow map. PROSAIL is a radiative transfer model, coupling a leaf optical property model (PROSPECT-D) and a canopy bidirectional reflectance model (4SAIL). The input parameter sets (p1, p2, p3) and reflectance of soils (s1, s2, s3) that are used by PROSAIL are presented in Table 2 and Figure S4, respectively. Synthetic datasets are generated with PROSAIL and used to develop random forest regression (RFR) models (Table S1). Three methods were tested, namely, (i) the “fIPAR method,” using Beer-Lambert Law to predict LAI from the fraction of intercepted photosynthetically active radiation (fIPAR) with setting extinction coefficient (*K*) to a constant (*K* =0.65 for wheat); (ii) the “RFR method,” using random forest regression models trained on synthetic dataset varying in parameter ranges (p1, p2, or p3) and soils (s1, s2, or s3) to predict LAI with plot-scale LAI retrieved from band reflectance map; (iii) the “RFR+LCB method” (“LCB” is the abbreviation for “Locally Calibrated Background”), using random forest regression models to predict LAI with plot-scale LAI retrieved from background-corrected band reflectance map. The application of “RFR method” and “RFR+LCB method” is illustrated in Figure 3.

The research flow map of this study is presented in Figure 1, consisting of the following steps. Three defined parameter sets were combined with specific soil reflectance to run PROSAIL to simulate canopy reflectance, resulting in several synthetic datasets. These synthetic datasets were used to train random forest regression (RFR) models to predict LAI from canopy reflectance. According to research objectives, these RFR models were firstly validated in subsets of synthetic data to investigate the effects of parameter range and soil background in theory. Subsequently, two proposed RFR model-based methods (“RFR method” and “RFR+LCB method”) (“LCB” is the abbreviation for “Locally Calibrated Background”) were validated on practical data from two field experiments and then compared with a baseline method (“fIPAR method”) based on Beer-Lambert Law.

### 2.2. Field Experiments

**Figure 2:**
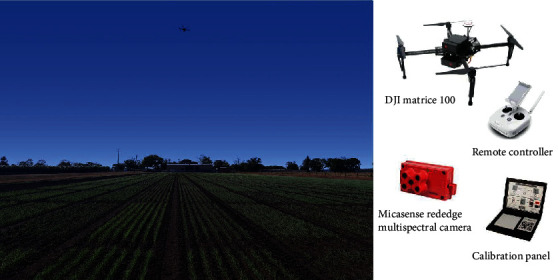
Phenotyping wheat trials with a UAV platform and key components for the UAV platform. The photo was taken at 11 : 50 a.m. on 31^st^ May 2019 (16 days after sowing).

Two wheat experiments were conducted at Gatton, Queensland (27.55°S, 152.33°E) in 2016 (Exp16) and 2019 (Exp19) and UAV-based phenotyping was undertaken along with field measurements (Figure 2). Different genotypes, plant densities, irrigation, and fertilization regimes created contrasting canopy structures in trials over an area of ~230 m by 50 m. Each experiment had four treatment blocks (with fillers to maintain physical isolation between blocks): irrigated and high nitrogen (IHN), irrigated and low nitrogen (ILN), rainfed and high nitrogen (RHN), rainfed and low nitrogen (RLN). Each block was split into small plots of ~14 m^2^ (2 × 7 m), each with 7 rows and a 25 cm row spacing. Weighed packets of seed adjusted for seed size and germination rate to achieve target plant density were sown by cone-seeder on May 21^st^, 2016 and May 15^th^, 2019, respectively. Fertilizer (50/205 kg ha^−1^ and 32.5/110 kg ha^−1^ for LN/HN in 2016 and 2019, respectively) was applied at sowing after measuring the preplanting soil nitrogen; irrigation was applied at sowing for all treatments for germination and was only applied after sowing for irrigated treatments when needed. Effective field practices were carried out to control weeds and diseases during the growing season. Plant emergence of all cultivars occurred approximately 10 days after sowing in 2016 and 5 days after sowing in 2019.

#### 2.2.1. Biophysical Measurements

Quadrat harvests comprised 0.5 m length of 4 inner rows (~0.5 m^2^). A subsample (25~33% of total biomass) was taken from the quadrat sample and partitioned into green leaves and other parts. Green leaves of the subsample were weighed (LFW_sub_, g) and then measured the leaf area (LA_sub_, cm^2^) with LI-3100C Area Meter. Subsequently, each part of a quadrat sample was placed in an oven at 70°C for 3-4 days and weighed to retrieve the oven-dry weight of green leaves of the subsample (LDW_sub_, g), subsample (DW_sub_, g), and quadrat sample (DW_main_, g). The LAI (m^2^ m^−2^), leaf water content (Cw, g cm^−2^), and leaf dry matter content (Cm, g cm^−2^) were calculated according to LAI = (LA_sub_ × DW_main_)/(QA × DW_sub_), Cw = (LFW_sub_ − LDW_sub_)/LA_sub_, and Cm = LDW_sub/_LA_sub_, where QA (cm^2^) is the ground area of quadrat harvest. The range of observed values of Cw, Cm, and LAI was used to limit the ranges of input parameters in the PROSAIL model when generating synthetic datasets (see details below). The light interception above (I_0_) and below (I_i_) canopy was measured with the AccuPAR LP-80 linear ceptometer (Decagon Devices, Pullman, WA) around noon (11 : 00 to 13 : 00) for all measurement dates to reduce the influence of solar zenith angle. The fraction of intercepted photosynthetically active radiation (fIPAR) was calculated according to fIPAR = 1 − I_i_/I_0_. For individual plots, the paired measurements of I_0_ and I_i_ were repeated three times by moving the ceptometer along the row direction to provide three instant measurements of fIPAR, which were averaged to provide a mean measured fIPAR for that plot. This mean fIPAR was used to estimate LAI based on a variant of Beer-Lambert Law, i.e., LAI = −ln (1 − fIPAR)/*K*, where extinction coefficient (*K*) was set to 0.65 for wheat according to a meta-analysis study about canopy light extinction coefficient [37]. This method was named as “fIPAR method,” which provided a baseline accuracy for LAI prediction.

Ground measurements were taken in 84 plots (including 7 genotypes, 1 density, 4 water-nitrogen treatments, 3 replicates) for Exp16, and 72 plots (including 3 genotypes, 3 densities, 4 water-nitrogen treatments, 2 replicates) for Exp19 (see green plots in Figure S3). Phenological observations once or twice per week recorded growing stages using a decimal code scale [38]. In Exp19, quadrat sampling to estimate traits such as LAI was taken with two harvests in all selected plots, and UAV phenotyping was performed the same day or two days before the quadrat harvest. In Exp16, quadrat harvest and UAV phenotyping did not always occur close to each other. Missing LAI measurements from quadrat harvest were interpolated with a fitted piecewise function based on all measured LAI from quadrat harvests across growth season (see Method S1; Figure S1). Finally, each selected plot had one ground-estimated LAI for each UAV-based estimate. This corresponded to a total of 252 and 144 estimated LAI values between plant emergence and flag leaf for Exp16 and Exp19, respectively (Table 1).

#### 2.2.2. Multispectral Data Collection and Processing

The multispectral data were captured from a UAV-based phenotyping platform from 10 : 00 to 14 : 00 at defined dates (Table 1) following protocols developed by Chapman et al. [24]. Five ground control points were evenly distributed in the field and fixed through the growing season. A mission plan for autonomous flights was designed to ensure 75% frontal overlap and 60% side overlap at least. The flight height for the three phenotyping dates in 2016 and the first date in 2019 was 20 m (corresponding to a 1.3 cm spatial resolution) and was 40 m (corresponding to a 2.7 cm spatial resolution) for the second flight in 2019. The multispectral camera used in this study was a MicaSense RedEdge camera (Figure 2; https://www.micasense.com) with 5 bands in the visible near-infrared range, i.e., Blue (475 nm centre wavelength, 20 nm bandwidth), Green (560 nm, 20 nm), Red (668 nm, 10 nm), Near-infrared (NIR) (840 nm, 40 nm), and Red edge (717 nm, 10 nm). Images of the calibrated reference panel from MicaSense were manually taken before and after each flight and then used for calibration in the later data processing. The UAV flights in two trials were finished within 30 minutes during which illumination was assumed to be stable in clear days without strong wind effects.

**Figure 3:**
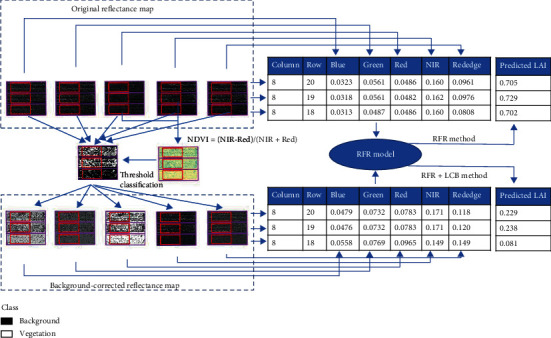
Schematics of processing images to provide input of random forest regression (RFR) model to predict LAI. Only information of three plots for Exp16 (DAS =18) are chosen here to simplify the illustration. The purple rectangles represent the full extent of the plot, and the red rectangles inside represent the extent of the trimmed plot. Two original reflectance maps (NIR and Red) are used to calculate the NDVI which is used to generate the vegetation-background binary map based on threshold classification. Using the binary map as a mask, the value of background pixels in the original reflectance map was replaced with the corresponding soil reflectance used in synthetic data, resulting in a new reflectance map named “background-corrected reflectance map.” The pixel-scale reflectance from the original reflectance map (“RFR method”) or the background-corrected reflectance map (“RFR+LCB method”) was averaged by trimmed plots (i.e., red rectangles) to generate the plot-scale reflectance that were used in RFR models to predict LAI.

After data acquisition, these raw images were processed (including geometric and radiometric correction) in Pix4Dmapper software (https://www.pix4d.com) to generate the ortho-image of calibrated reflectance of each band for the whole field. Two reflectance bands (NIR and Red) are used to calculate the NDVI which is used to generate the vegetation-background binary map based on threshold classification. The threshold was empirically set as 0.5 (tillering stage), 0.65 (stem elongation stage), and 0.75 (flag leaf stage). These threshold values can effectively classify soil pixels as background and avoid classifying green leaves as background, especially when plants are small. Using the binary map as a mask, the value of background pixels in the original reflectance map was replaced with the band value of corresponding soil reflectance used in the specific synthetic dataset, resulting in a new reflectance map named “background-corrected reflectance map.” Further details about this classification method and background correction are provided in the supplementary materials (Method S2). The entire field extent was segmented into individual plots according to the experimental design. Individual plots were trimmed by a percentage of 10% along four sides to exclude marginal areas from adjacent plots and plot gaps. The harvested areas were also clipped from the plot images. The pixel-scale reflectance from the reflectance map was averaged by trimmed plots to generate the plot-scale reflectance that were used in RFR models to predict LAI. There are two RFR model-based methods—“RFR method” and “RFR+LCB method” (“LCB” is abbreviation for “Locally Calibrated Background”). They use the same RFR model trained over synthetic dataset to predict LAI. The difference is that “RFR method” predicts LAI from the plot-level reflectance aggregated from the original reflectance map, while the plot-level reflectance was aggregated from the background-corrected reflectance map in “RFR+LCB method.” The flow of processing reflectance map to generate plot-scale reflectance for LAI prediction in two methods is illustrated in Figure 3 with a few plots as example.

### 2.3. Simulating Synthetic Datasets with the PROSAIL Model

The PROSAIL model couples a leaf optical property model (PROSPECT [39]) and a canopy bidirectional reflectance model (SAIL [40]), which can be used to estimate canopy variables such as LAI from canopy reflectance [36]. The current version of PROSAIL, used to simulate canopy reflectance in this study, is PROSAIL-D (PROSPECT-D coupled with SAIL) (http://teledetection.ipgp.jussieu.fr/prosail/). PROSAIL takes as input the soil reflectance (Figure S4) as well as parameters related to the following (Table 2): (i) leaf properties; (ii) canopy architecture; (iii) soil adjustment factor; (iiii) solar-object-sensor observation geometry. The fraction of diffuse illumination (*skyl*) was adjusted based on solar zenith angle in the current version of PROSAIL, though others have fixed it to a constant value (e.g., *skyl* =0.1 in [8, 13], *skly* =0.2 in [41]). The model outputs multiple canopy reflectance from 400 to 2500 nm at 1 nm interval and here only bidirectional reflectance was used for analysis, which was resampled into band reflectance of the five bands based on spectral response coefficient provided by MicaSense (see Method S3; Figure S2).

In this study, three varying sets of PROSAIL input parameter ranges were considered to generate synthetic datasets (Table 2). Set#1 represented a very broad range of parameter values for leaf and canopy properties of wheat, which were summarized in a review paper [42]. In Set#2, parameter inputs for Cm, Cw, and LAI were limited to narrower ranges based on observed values from Exp16 and Exp19. Sensitivity analysis of PROSAIL indicated that canopy reflectance in NIR range can be affected by variation of LAI or Cm alone but not Cw [8], but that interactions among parameters can also alter canopy reflectance in NIR range [43]. Calculating the difference of canopy reflectance caused by changing Cm from 0.001 to 0.01 at specific levels of Cw (i.e., 0.01, 0.02, 0.03) by holding other parameters to their average values, our results showed that the difference of canopy reflectance in NIR range increased with increasing Cw level and independent to soil background (Figure S5). The range of LAI values in Set#3 was further limited to LAI range of 0-5 m^2^ m^−2^ as (i) LAI rarely exceeds 5 m^2^ m^−2^ in most situations in the Australian wheatbelt, and (ii) LAI estimation based on reflectance are difficult for closed canopies with high LAI in natural conditions or even in simulations (Figure S6). Overall, observed LAI was less than 5 m^2^ m^−2^ for over 90% of the samples in the two field experiments (i.e., 99% in Exp16 and 93% in Exp19). *VZA* was fixed to zero as the images were captured under nadir view and camera distortion correction was applied in image processing to obtain orthoimages. The ranges of *SZA* and *RAA* were determined based on practical UAV phenotyping time (i.e., 10 : 00 to 14 : 00), day of year throughout the potential wheat growth season (April to December), and the range of latitudes across the Australian wheatbelt. There are two additional considerations: (i) the value of *hspot* defined here is only used when *VZA* is equal to *SZA* in which condition the hspot effect occurs; otherwise, hspot is reset to 0; (ii) the *RAA* is equal to *SAA* when *VZA* = 0 in which condition the changes of *RAA* will not alter reflectance, and *RAA* can be directly set to a constant value.

The reflectance of soil background is determined by the local geology and the wetness of the soil. In this study, the soil background was assumed to be a Lambert reflector (with the same reflectance independent to viewing angles) as the prediction of LAI would be made with the RFR model trained over synthetic dataset generated from PROSAIL (see details below). In PROSAIL, an adjustment parameter (*psoil*), ranging from 0 to 1, is used to adjust the soil reflectance based on soil wetness. PROSAIL provides a default soil background, with standard soil reflectance under wet and dry conditions. The soil reflectance for two field experiments was measured with an ASD FieldSpec Spectroradiometer (https://www.malvernpanalytical.com/asd) at sowing dates before (dry condition) and after (wet condition) irrigation, and spatial variations of soil background were assumed to be negligible based on inspection of variation in exposed soil reflectance in alleyways and paths across the field. To simplify the model parameterization, the *psoil* was fixed to 1 through all simulations as the soil surface was dry at actual flight times. The reflectance of three soil types was used in this study for generating synthetic datasets. Those were (i) the default soil from PROSAIL and measured soil reflectance for (ii) Exp16 and (iii) Exp19 (Figure S4).

To make our proposed LAI estimation method more generic to potential soil types and independent to ASD data, we investigated the possibility to retrieve soil characteristics from bare soil pixels in the multispectral images. In addition to the wetness factor (*psoil*) used to mix the wet and dry soil, the possible variation of soil background reflectance can be accounted for by a multiplicative brightness factor (*asoil*) [42]. Thus, the reflectance of a particular soil (*Rsoil*) with specific wetness and brightness relative to the default soil provided in the PROSAIL model can be calculated as:

(1) $$\begin{array}{c} Rsoil=asoil\times \left(ps\text{o}il\times Rsoil_{dry}+\left(1-psoil\right)\times Rsoil_{wet}\right), \end{array}$$

where *Rsoil*_*dry*_ and *Rsoil*_*wet*_ represent the reflectance of default soil under dry and wet conditions, respectively. Based on this hypothesis, we proposed an approach to estimate the *psoil* and *asoil* by minimizing the objective function (f) below:

(2) $$\begin{array}{c} f=\min\left\{\sum \left(asoil\times \left(psoil\times Rsoil_{dry\left(i\right)}+\left(1-psoil\right)\times Rsoil_{wet\left(i\right)}\right)-Rsoil_{obs\left(i\right)}\right)\right\}, \end{array}$$

where *Rsoil*_*dry*(*i*)_, *Rsoil*_*wet*(*i*)_, and *Rsoil*_*obs*(*i*)_ represent the default dry soil reflectance, the default wet soil reflectance, and the observed soil reflectance of the *i*^th^ band of sensors and cameras. With the proposed soil calibration approach, the simulated soil reflectance (with 1-nm interval in 400-2500 nm) of two experiments was provided by adjusting default soil reflectance based on Eq. (1) with fitted values of *asoil* and *psoil*, which were obtained by minimizing the objective function Eq. (2) between resampled band reflectance of default soil and observed band reflectance retrieved from bare soil pixels of UAV multispectral images.

A subset consisting of 40000 combinations of PROSAIL input parameters was randomly sampled from the defined parameter spaces of Set#1, Set#2, and Set#3 (Table 2). These input combinations were then combined with specific soil reflectance to run PROSAIL to simulate canopy reflectance. Nine datasets corresponding to the reflectance of five bands were generated for the three parameter sets (p1, p2, and p3 for Set#1, Set#2, and Set#3, respectively) and the reflectance of three types of soil (s1, s2, and s3 for the soils of the default, Exp16, and Exp19, respectively). The nine datasets are named as Dp1s1, Dp1s2, Dp1s3, Dp2s1, Dp2s2, Dp2s3, Dp3s1, Dp3s2, and Dp3s3. Distinguished with soil reflectance measured with ASD in two experiments (i.e., s2 for Exp16, s3 for Exp19), the simulated soil reflectance retrieved with the proposed soil calibration approach was named as s2∗ for Exp16 and s3∗ for Exp19, respectively. These two types of simulated soil reflectance were then used to generate another six synthetic datasets (i.e., Dp1s2∗, Dp1s3∗, Dp2s2∗, Dp2s3∗, Dp3s2∗, Dp3s3∗) (used for training models) to verify this calibration approach's applicability in practical LAI estimation.

### 2.4. Estimations of LAI with Random Forest Regression Models

#### 2.4.1. Model Training

Random forest (RF) is an ensemble machine learning method based on decision tree algorithms. The final prediction is the average of predictions from multiple decision trees (base learners), trained on different subsets of the same dataset with the aim of minimizing overfitting by individual base learners [44]. Here, for the model training, the predictive variables of a random forest regression (RFR) model are the reflectance of five bands and LAI is the response variable. Both reflectance and LAI were normalized with the zero-mean normalization approach to prevent any scaling issues. The mean squared error (MSE) was used to evaluate the model performance during training. Each dataset was randomly split into two subsets: 75% (30,000 samples) as training set and 25% (10,000 samples) as test set. For clarity, models were named as the dataset used for training with, e.g., “Mp1s1” representing the model trained on “Dp1s1” dataset (Table S1).

To increase the inter-individual differences between base learners, bootstrap sampling was used to add sample disturbance and the maximum feature number was set to “max_features = log2(n_features)” to add attribute disturbance (here n_features = 5, corresponding to the five band reflectance). A set of values for (i) the number of base learners (n_estimators of 5, 50, 100, 200, 300, 400, 500, 1000) and the minimum number of samples required to be at a leaf node (min_samples_leaf of 1, 5, 10, 15) were tested to find the optimal hyperparameter combination of RFR. The hyperparameter tuning was only conducted on training model “Mp1s1” and the selected optimal hyperparameter combination was used for all other 8 models in later analysis. During the process of tuning hyperparameters, the training set (“Dp1s1”) was randomly divided into training and tuning set and 3-fold cross-validation was conducted, which indicated model performance reached stability when n_estimators was up to 200, with min_samples_leaf = 1 resulting in the best accuracy (over the tuning set). The RFR model was implemented with Python 3.7.2 using the scikit-learn open-source machine learning library (V 0.24.2, https://scikit-learn.org/stable/).

#### 2.4.2. Model Evaluation

The RFR models trained on synthetic datasets (as shown in Section 2.3.1) were used to make predictions (i) for “out-of-sample” synthetic data (i.e., the test sets) to evaluate the theoretical performance and (ii) for experimental data to evaluate the practical performance (Table S1).

A major aim of phenotyping in breeding is to capture the changing pattern of the LAI curve over time as well as variations in LAI caused by varying genotypes and management practices. Thus, correlation coefficient was used to evaluate model performance, i.e., Pearson's correlation coefficient (*r*) to evaluate the correlation between the observation and its prediction, and Spearman's rank correlation coefficient (*r*_s_) to evaluate the rank correlation. Pearson's correlation coefficient (*r*) indicates the degree to which the movement of observed (or known) LAI is captured in predicted LAI, while the Spearman rank correlation coefficient (*r*_s_) tells the rank correlation between them. The determination coefficient (*R*^2^) measures the ability of RFR model to predict LAI, indicating the proportion of the variance in observed (or known) LAI can be explained by predicted LAI in the linear regression setting. Both root mean squared error (RMSE) and RRMSE (a ratio of RMSE divided by the mean of observed (or known) LAI) measure the prediction accuracy of RFR model, indicating the average absolute and relative error between the known (or observed) LAI and its prediction retrieved from RFR model, respectively. All metrics were calculated in R 3.6.0.

Each type of synthetic dataset (used to train RFR model) was sampled from the entire parameter space multiple times, resulting in varying subsets equivalent to “replicates” (each with 40,000 samples). Only results from the first sampling were presented below as models trained on different replicates had very similar performances (data not shown).

## 3. Results

### 3.1. Model Theoretical Performance of LAI Estimation on Synthetic Data

**Figure 4:**
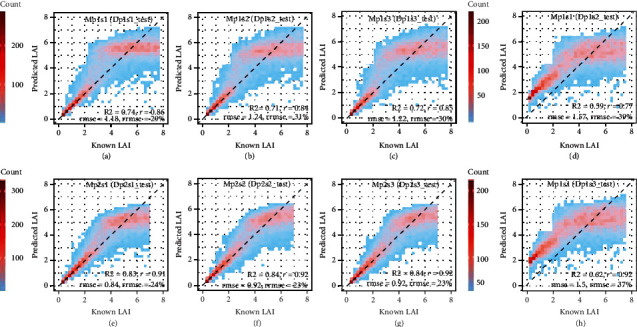
Known LAI against LAI predicted with RFR models trained on synthetic datasets for respective theoretical test datasets (*n* =10,000) (Table S1). Each model was trained and tested on independent subsets of the synthetic dataset that was produced by PROSAIL with a specific parameter range (p1, p2) and soil (s1, s2, s3). Known LAI corresponding to input LAI values used to run PROSAIL and generate the synthetic datasets.

The theoretical performance of RFR models trained on synthetic PROSAIL datasets was first evaluated for the synthetic test set (i.e., out-of-sample set). Theoretical performance of models trained on synthetic datasets with varying soil reflectance (i.e., Mp1s1, Mp1s2, and Mp1s3) was very similar when evaluated for independent subsets of the synthetic data they were trained for, with *r* of 0.84~0.86, *R*^2^ of 0.71~0.74, RMSE of 1.18~1.22 m^2^ m^−2^, and RRMSE of 29~31% (Figures 4(a)–4(c)). Less accurate estimations were found when testing models with test sets from other soils, with additional overestimation for LAI <2 (Figures 4(d) and 4(h)) in which range it was less likely to be overestimated if testing on the same soil (Figure 4(a)). For instance, *R*^2^ of “Mp1s1” was reduced from 0.74 for the same soil (Figure 4(a)) to 0.59~0.62 for other soils (Figures 4(d) and 4(h)). This indicates that, theoretically, RFR models can achieve good estimation accuracy of LAI for different soil types. Narrowing parameter ranges (replacing p1 with p2) increased *r* from 0.84~0.86 to 0.91~0.92, increased *R*^2^ from 0.71~0.74 to 0.83~0.84, reduced RMSE from 1.18~1.22 m^2^ m^−2^ to 0.84~0.92 m^2^ m^−2^, and reduced RRMSE from 29~31% to 23~24% (Figures 4(e)–4(g)). All models tended to overestimate LAI for 2 < LAI < 5 and underestimate LAI for LAI > 5 irrespective of the parameter set or soil used.

The model performance presented in Figure 4 was further evaluated in more detail for three LAI levels: LAI ≤ 2, 2 < LAI ≤ 5, and LAI > 5 (Figure S7). As expected, the RMSE increased with increasing values in LAI level, while the RRMSE decreased with increasing values in LAI level. The values of *r* and *R*^2^ decreased when LAI changed from “LAI > 2” to “2 < LAI ≤ 5” and dropped to a low value (*r*<0.2, *R*^2^<0.05) when LAI changed to “LAI > 5.” Hence, the RFR model has a low probability to correctly predict LAI for LAI > 5, even though the estimation error remained relatively small.

An overestimation of LAI was observed for 2 < LAI ≤ 5 (Figure 4). Such overestimation was likely due to that the RFR model tried to compensate for the underestimation of LAI for LAI > 5 to achieve a global optimization during the training phase. To test this hypothesis, LAI values were only considered up to 5 m^2^ m^−2^ in models Mp3s1, Mp3s2, and Mp3s3. These models were compared to the equivalent models Mp2s1, Mp2s2, and Mp2s3 that considered input LAI up to 7 m^2^ m^−2^. Results indicated that Mp2s1 outperformed Mp3s1 on the same test set (Dps3s1_test, 0 < LAI ≤ 5), with similar *r*, smaller RMSE and RRMSE, and more samples close to the 1 : 1 line (Figure S8a, b). The overall improvement for 0<LAI≤5 was mainly achieved by improving LAI estimation in the range of 2-4, without no apparent improvement for LAI<2, while the estimation of LAI in the range of 4-5 changed from overestimation to underestimation (Figure S8c, d). Similar results were found for the other two tested soils (i.e., Mp2s2 vs Mp3s2; Mp2s3 vs Mp3s3) (Figure S8(e)–S8(i)).

### 3.2. Overall Estimation of LAI for Different Field Experiments

The performance of the RFR models was also tested against experimental data (Figure 1). Firstly, RFR models trained on synthetic datasets generated with “default soil” (i.e., Mp1s1, Mp2s1, and Mp3s1) were tested against data from both experiments Exp16 and Exp19. Secondly, models trained on synthetic datasets generated with Exp16 soil (i.e., Mp1s2, Mp2s2, and Mp3s2) were tested against Exp16 data; and thirdly, models trained on synthetic datasets generated with Exp19 soil (i.e., Mp1s3, Mp2s3, and Mp3s3) were tested against Exp19 data.

**Figure 5:**
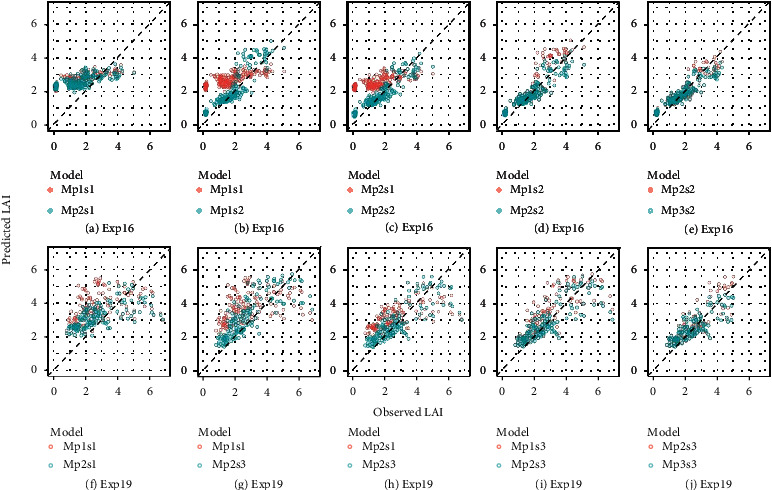
Observed LAI against predicted LAI for Exp16 (a, b, c, d, e) and Exp19 (f, g, h, i, j) from “RFR” method. In (a, d, f, i), the two models in the same subfigure were trained on synthetic datasets varying in parameter range: red symbols correspond to a wider range (p1), while blue symbols correspond to a narrower range (p2). In (b, c, g, h), the two models in the same subfigure were trained on synthetic datasets varying in soil characteristics: red symbols correspond to default soil from the PROSAIL model (s1), while blue symbols correspond to soil measured during the experiments (s2, s3). In (e, j), the two models in the same subfigure were trained on synthetic datasets varying in LAI range: red symbols correspond to PROSAIL input LAI values ranging from 0 to 7 m^2^ m^−2^ (p2), while blue symbols correspond to PROSAIL input LAI range of 0-5 m^2^ m^−2^ (p3). Exp16 had 252 data points (251 data points with LAI≤5) and Exp19 had 144 data points (130 data points with LAI≤5). All statistical metrics are summarized in Table 3.

Practical performance of RFR models on experimental data (with original reflectance), also referred as the “RFR method,” is shown in Figure 5 and Table 3. Without correcting soil reflectance (i.e., using the default soil s1), model Mp1s1 could not systematically make a good estimation of LAI from canopy reflectance captured in the field, with a good correlation for Exp16 (*r*=0.79), a poor correlation for Exp19 (*r*=0.43), and a systematic overestimation of LAI especially for LAI<2 (Figures 5(a) and 5(f); Table 3). The estimation of model accuracy could be improved by limiting ranges of some key parameters (i.e., Cm, Cw, LAI) based on field measurements when generating synthetic datasets: value of *r* for Exp19 increased from 0.43 (Mp1s1) to 0.64 (Mp2s1) (Figure 5(f); Table 3). Replacing p1 with p2 could systematically reduce estimation error in LAI range of 0-7 (shifting datapoints down closer to 1 : 1 line) but could not effectively correct the overestimation for low LAI (LAI<2) (Figures 5(a) and 5(f)). In contrast, correcting soil reflectance with local soil characteristics appeared to be a valid means to improve models' performance, as this substantially increased *r* and decreased RMSE for two both experiments (Table 3), especially during early development when crops only cover a small part of the soil (Figures 5(b), 5(c), 5(g), and 5(h)). Given soil reflectance in the synthetic dataset was corrected with local soil data, the improvements via limiting parameter ranges were limited (Figures 5(d) and 5(i); Table 3).

Analysis of synthetic data from Section 3.2 indicated that narrowing LAI range to 0<LAI≤5 could effectively improve LAI estimation in the range of 2-4 (Figure S8). Consistent with theoretical results, RFR models trained with synthetic datasets with LAI in 0<LAI≤5 rather than 0<LAI≤7 could result in more accurate estimation for LAI in the range of 2-4 for experimental data, without no apparent improvements for estimation of LAI below 2, but with underestimation for LAI above 4 (Figures 5(e) and 5(j)). However, these improvements on experimental data were less obvious than theoretical improvements on synthetic data.

### 3.3. Comparing Estimation Accuracy of LAI Predicted with Varying Methods

**Figure 6:**
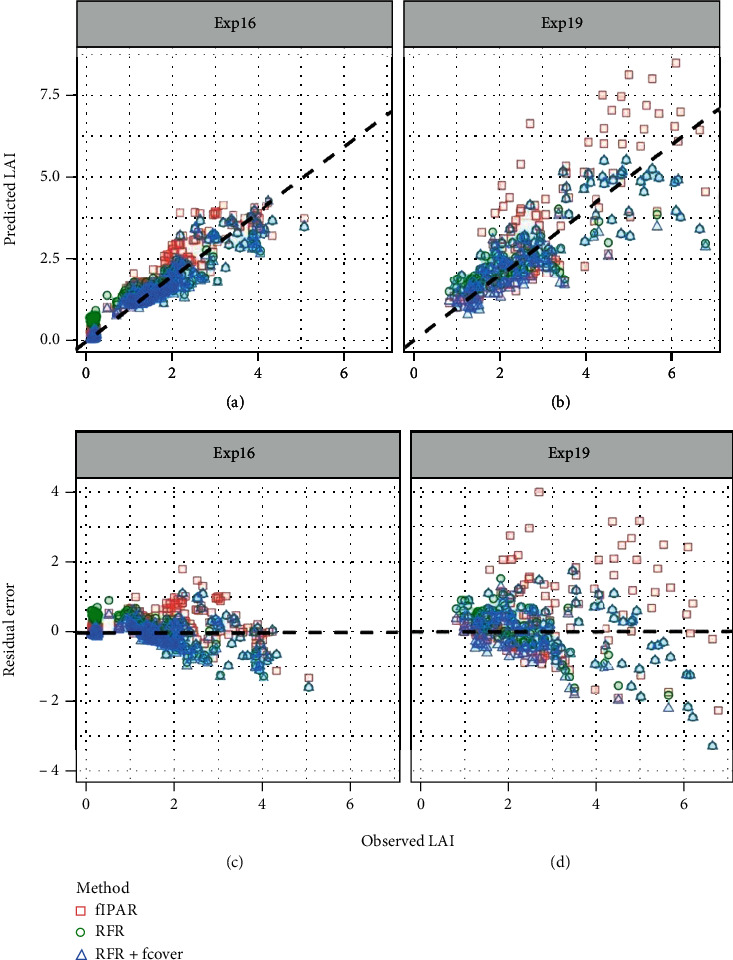
Observed LAI against predicted LAI (a, b), and observed LAI against predicted residual (c, d). LAI was predicted with three methods, i.e., Method “fIPAR,” using Beer-Lambert to predict LAI from the fraction of vegetation with setting *K* to a constant (here *K* =0.65 for wheat); Method “RFR,” using random forest regression models trained on synthetic datasets to predict LAI from original reflectance; Method “RFR+LCB,” the same RFR model to predict LAI from background-corrected reflectance. RFR models used in “RFR method” and “RFR+LCB method” were Mp2s2 and Mp2s3 for Exp16 and Exp19, respectively. Residual error corresponds to the difference of observed LAI subtracted from predicted LAI. All statistical metrics are summarized in Table 3.

The values of LAI predicted with “RFR method” were compared with corresponding values predicted with “fIPAR method” and “RFR+LCB method” (Figure 6; Table 3). The “fIPAR method” was not able to systematically make accurate estimations of LAI for two field experiments, with a large range of residual error ranging from -3 to 4 m^2^ m^−2^ for Exp19 (Figure 6). In contrast, the “RFR” method could more robustly achieve accurate estimation of LAI up to 5 m^2^ m^−2^ (Figure 6), reducing residual error in the range from -2 to 2 m^2^ m^−2^ for both field experiments (Table 3). Applying background correction on original reflectance map, the “RFR+LCB method” further improved estimation accuracy of LAI through improving estimation of LAI below 2 m^2^ m^−2^ (Figure 6), reducing RMSE and RRMSE of 0.1 m^2^ m^−2^ and 8% for Exp16 (Table 3).

Overall, these findings indicated that a constant value of extinction coefficient (*K* =0.65) was insufficient to accurately predict LAI in field conditions, as *K* was site-specific and sensitive to environmental changes (Figure S9). By contrast, the “RFR method” could effectively predict LAI at plot level under the field conditions of this study (Figure S10), given RFR models were trained on synthetic datasets with local soil background (s2 for Exp16, s3 for Exp19) regardless of parameter sets (p1, p2, p3). However, for sparse canopy with soil being obviously exposed (e.g., wheat canopy at tillering stage in which plants are small), the background correction of reflectance map is needed to achieve accurate LAI estimation as presented in “RFR+LCB method” which requires accurate classification of vegetation and background. The “RFR method” can achieve accurate estimation for LAI up to 7 m^2^ m^−2^ (*r*=0.80, *R*^2^=0.64, RMSE=0.84 m^2^ m^−2^, RRMSE=30%) for Exp19, while the accurate estimation of LAI (*r*=0.95, *R*^2^=0.91, RMSE=0.36 m^2^ m^−2^, RRMSE=25%) for Exp16 was achieved from “RFR+LCB method” (Table 3).

### 3.4. Mapping Spatiotemporal Variation of LAI for Different Phenotyping Dates

**Figure 7:**
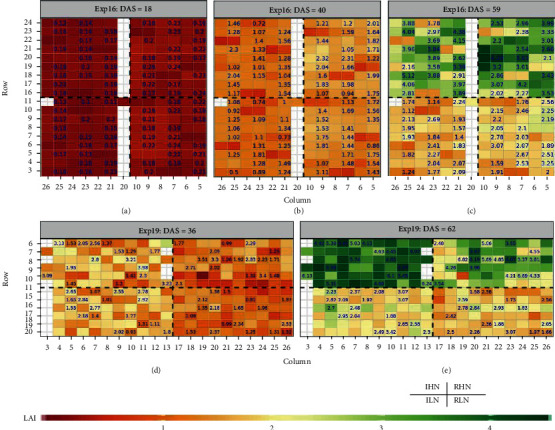
Predicted LAI retrieved with “RFR+LCB method” from UVA-based multispectral images on different dates for two experiments. The RFR models used “RFR+LCB method” were Mp2s2 and Mp2s3 for Exp16 and Exp19, respectively. The number shown on the top of the map indicates the observed LAI for the specific plot on the corresponding date. For each subfigure, only 84 (for Exp16) and 72 (for Exp19) plots have observed LAI as biophysical measurements were only conducted in these plots (referring to Figure S3). Row and Column are used to locate the position of the plot in the field.

The predicted LAI retrieved with “RFR+LCB method” for each plot within the four blocks is shown in Figure 7, which presents the spatiotemporal variation of plot-scale LAI during different growing stage for two experiments. Within each map, the four blocks correspond to the four water-nitrogen treatments, i.e., IHN (top left), RHN (top right), ILN (bottom left), and RLN (bottom right). The predicted LAI accurately captures the increasing trend of observed LAI along with time from tillering to flag leaf stages for Exp16 (Figures 7(a)–7(c)) and from start elongation to flag leaf stages for Exp19 (Figures 7(d) and 7(e)). Additionally, the predicted LAI clearly presents the difference in observed LAI under different treatments and how this spatial pattern changes with time. At the tillering stage, there is no obvious difference in LAI among four treatments (Figure 7(a)). At the elongation stage, effects of different water treatments can be captured in predicted LAI: values of predicted LAI appear slightly higher under rainfed treatment than under irrigated treatment for Exp16 as the high rainfall in early season (Figure 7(b)) while LAI is lower under rainfed treatment than under irrigated treatment for Exp19 (Figure 7(d)). At the flag leaf stage, effects of different nitrogen treatments as well as water treatments can be identified simultaneously: values of predicted LAI under high-nitrogen treatments are higher than those under low-nitrogen treatments for both Exp16 (Figure 7(c)) and Exp19 (Figure 7(e)). All comparisons were significant at 0.05 level. The statistical metrics between predicted and observed LAI for different experiments can be found in Table 3 and detailed analysis for different treatments will be demonstrated in the following sections.

### 3.5. Predicting LAI Differences within Growing Stages or Treatments

**Figure 8:**
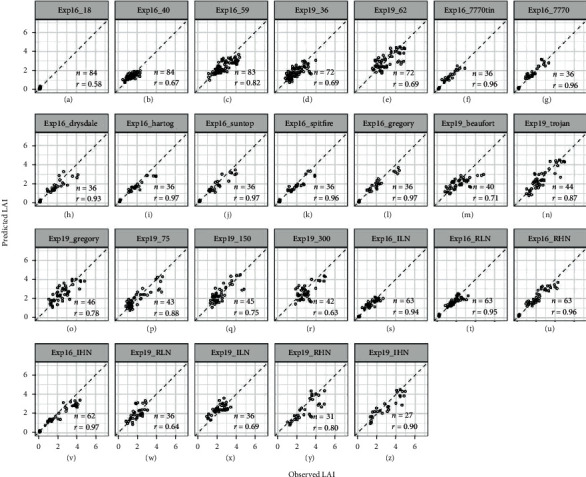
Observed LAI against predicted LAI for two field experiments (observed LAI<=5) for different growing stages (a–e), genotypes (f–o), plant densities (p–r), and other management practices (s–z). Model Mp3s2 and Mp3s3 were used in “RFR+LCB method” to predict LAI for Exp16 and Exp19, respectively. Pearson correlation coefficients (*r*) between observed and predicted LAI are given for each group (e.g., DAS =18, Density = 75) within an experiment. All statistical metrics are summarized in Table S2.

In this section, model performance was segmented by growing stages to identify (or predict) the intra-factor difference for different growing stages (Figures 8(a)–8(e)), i.e., investigate LAI differences across varying genotypes and treatments at specific growth stages. Likewise, segmentation analyses were performed for factors of genotype, density, and water-nitrogen treatments (Figures 8(f)–8(z)). Only performance was evaluated for “RFR+LCB method” with Mp3s2 (predicting on Exp16) and Mp3s3 (predicting on Exp19), which theoretically overcome the overestimation of LAI in 2<LAI<5. Correlation coefficient (*r*) was used here for evaluation on using over 90% samples from field experiments in order to evaluate if the movement of observed LAI was well captured in predicted LAI retrieved from RFR models (results of other metrics like *R*^2^, RMSE, and RRMSE referring to Table S2).

There was an increased *r* between observed and predicted LAI from tillering (*r* = 0.58, Figure 8(a)), stem elongation (*r* = 0.67, Figure 8(b)), to flag leaves (*r* = 0.82, Figure 8(c)) for Exp16, while the *r* was relatively stable from stem elongation (*r* = 0.69, Figure 8(d)) to flag leaves (*r* = 0.73, Figure 8(e)) for Exp19. This indicated our method was not sensitive enough to distinguish the relative difference in LAI among different treatments (i.e., genotype, density, water-nitrogen application) at the very beginning of growth development (e.g., tillering, LAI<0.5), as models' prediction error might be larger than the variation of observed LAI caused by treatments. The higher planting density for Exp19 resulted in more centralized distribution of LAI to high values, which reduced the variation of observed LAI caused by other factors, leading to a decreasing performance of our method (with *r* reducing from 0.88 to 0.75 and 0.63 when increasing density from 75 to 150 and 300) (Figures 8(p)–8(r)). For Exp16, our method could correctly predict the intra-factor difference for different genotypes (Figures 8(f)–8(o)) or different water-nitrogen managements (Figures 8(s)–8(z)), with a high *r* above 0.93 between observed and predicted LAI under all situations. However, the intra-factor difference for Beaufort (Figure 8(m)) or low nitrogen managements (Figures 8(w) and 8(x)) was not well distinguished for Exp19. In addition, the RRMSE achieved from segment analysis under all specific situations was relatively stable, varying in 21~33% depending on situations analyzed (Table S2).

### 3.6. Predicting LAI Differences among Growing Stages or Treatments

**Figure 9:**
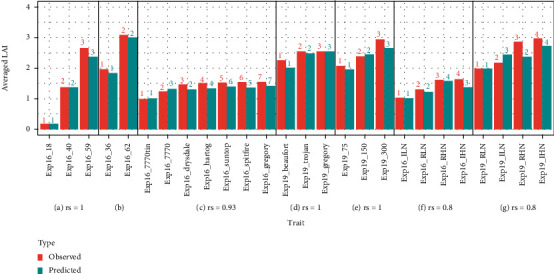
The averaged observed LAI (coloured in blue) and averaged predicted LAI (coloured in red) on experimental data (observed LAI<=5) for different growing stages (a, b), genotypes (c, d), plant densities (e), and other management practices (f, g). Here, Mp3s2 and Mp3s3 were used in “RFR+LCB method” to predict LAI for Exp16 and Exp19, respectively. For each group (e.g., DAS, Density), the number on top of each bar indicates the rank of the LAI value averaged for a specific group level within the group (e.g., DAS =18, Density = 75) within each experiment. The “*r*_s_” represents the rank correlation coefficient between average observed LAI and average predicted LAI for a specific group (e.g., DAS, Density) within an experiment.

Following segmentation analyses for intra-factor difference, this section focused on evaluating the performance of the same two models (i.e., Mp3s2 and Mp3s3) for “RFR+LCB method” in predicting inter-individual difference for different growing stages (Figures 9(a) and 9(b)), i.e., investigate the rank of averaged LAI among varying growing stages due to the cumulative effects of various treatments. Likewise, rank correlation analyses were performed for varying levels of genotype, density, and water-nitrogen treatments (Figures 9(c)–9(f)).

The rank of averaged predicted LAI for varying growing stages for two experiments was the same as those of average observed LAI (Figures 9(a) and 9(b)), indicating our method could correctly detect dynamic changes of LAI at early stages (i.e., from tillering to flag leaf) under cumulative effects of various treatments. The ranks of averaged observed LAI among varying density treatments in Exp19 were also predicted completely correctly (Figure 9(e)). However, our method failed to predict the rank of averaged observed LAI for some genotypes for Exp16 (Figure 9(c), between which the average observed LAI was not significantly different at 0.05 significant level (Table S3). Our method could not completely predict the rank of averaged observed LAI for water-nitrogen treatments either (Figures 9(f) and 9(g)), which was due to the insignificant difference of averaged observed LAI between RHN and IHN for Exp16 (Figure 9(f); Table S3) and due to the inconsistent prediction tendency between ILN and RHN (i.e., overestimation for ILN (Figure 8(x)) and underestimation for RHN (Figure 8(y))) for Exp19 (Figure 9(g)). In other words, our method could correctly predict the rank of averaged LAI for varying group levels of a specific group, given the average values of observed LAI were significantly different and prediction tendency was consistent among levels.

### 3.7. Application of Simulated Soil Characteristics Retrieved from Multispectral Images

To demonstrate the simulation accuracy of the proposed soil calibration approach, it was first applied to simulate soil reflectance of two trials based on soil band reflectance resampled from ASD data. The simulated band reflectance fitted with the proposed soil calibration approach was highly correlated with actual band reflectance measured with ASD (Figure S11(a)), with a relative bias for all bands within 5% except for the blue band (16%) (Figure S11(b)). This indicated that this proposed approach could provide a simulated soil reflectance approximated to the actual soil reflectance of targeted bands. Subsequently, this approach was used to simulate soil reflectance based on soil band reflectance retrieved from bare soil pixels of UAV multispectral images. Likewise, the simulated soil reflectance for all bands was highly correlated with their observations retrieved from UAV images (Figure S11(c)). In addition, the simulated reflectance in 400-900 nm, based on fitted soil factors (i.e., *asoil* and *psoil*) retrieved from UAV images, was alike for the two trials and slightly lower than their counterparts measured on the ground with ASD FieldSpec (Figure S11(d)). The high similarity of the two simulated soil reflectance was mainly due to their observations for five bands being quite similar (especially from UAV images) and partly resulted from the fitness errors.

Corresponding to soil reflectance measured with ASD (s2 for Exp16, s3 for Exp19), the simulated soil reflectance retrieved from UAV images with the proposed approach was named as s2∗ for Exp16 and s3∗ for Exp19. The estimation accuracy of LAI predicted with models trained over synthetic datasets with simulated soil reflectance is presented in Figure S12 and Table 3. Compared with estimation accuracy of LAI predicted with models using measured soil reflectance, the models using simulated soil reflectance achieved similarly high accuracy for LAI estimation independent of the use of parameter sets (p1, p2, p3), especially with “RFR+LCB method” (Table 3). In particular, the models using simulated soil reflectance resulted in even smaller errors for estimation of LAI under 2 m^2^ m^−2^ in “RFR” method (Figure S12(a), S12(c), S12(e)). This was likely due to the simulated soil reflectance being more similar to that soil reflectance captured from UAV, as the simulated soil reflectance was calibrated with the reflectance of soil pixels from UAV images while measured soil reflectance was collected on the ground with ASD.

## 4. Discussion

### 4.1. A Method to Estimate LAI Using Canopy Reflectance

Our study proposed a method, named here “RFR+LCB,” which enables accurate estimation of LAI particularly in the range from 1 to 5, from canopy reflectance in field conditions. Three factors are mainly affecting the practical performance of the method, i.e., parameter ranges, soil reflectance, and vegetation/background binary classification. Narrowing parameter ranges (changing p1 to p2) theoretically allows parameter combinations changing in a smaller subset of the original parameter space, which reduced the problem complexity and then resulted in our study in better model performance (Figure S7). In practice, approximating ranges of parameters to their actual ranges can increase the similarity of data distribution between training synthetic data and experimental data. Thus, models trained on synthetic data presented were improved when narrowing the parameter ranges according to the studied experimental conditions: p2 models outperformed p1 models for estimation of LAI up to 7 m^2^ m^−2^, while p3 models outperformed p2 models for estimation of LAI up to 5 m^2^ m^−2^ (Figure 5; Table 3). This conclusion was confirmed in other hybrid methods in which LAI estimation was improved from limiting parameter ranges to field observations [31, 45, 46]. However, the effects of parameter ranges on estimation accuracy were relatively small compared to the other two factors (Table 3), as these three parameter sets were within the general range of corresponding traits of wheat (Table 2).

The setting of soil reflectance also affects the similarity between training synthetic data and experimental data, since simulated canopy reflectance is co-determined by leaf and soil reflectance in PROSAIL and soil reflectance serves as the lower boundary [36]. Soil reflectance contributes more to canopy reflectance for sparse canopies than for dense canopies [43, 47], as shown also in our study, i.e., replacing default soil reflectance (s1) with measured soil reflectance (s2, s3) for synthetic dataset generation resulted in larger improvement of LAI estimation for LAI<2 than for LAI>2 in two field experiments (Figure 5(b), 5(c), 5(g), and 5(h); Table 3). This finding was consistent with an early theoretical study which also reported sensitivity of canopy reflectance to soil background was not ignorable for LAI<2 and but had small effects for LAI>2 [41]. Although the importance of soil reflectance in simulating canopy reflectance is well known, the soil reflectance spectra used in PROSAIL were rarely mentioned in previous studies, which rarely considered LAI less than 2 m^2^ m^−2^ [32]. However, the LAI rarely exceeds 5 m^2^ m^−2^ in most situations in the Australian wheatbelt in which the wheat canopy has LAI<2 for at least one month (as in our study). In sandy soils, the LAI may rarely exceed 2 m^2^ m^−2^ during the whole growing season under low rainfall rainfed conditions, e.g., at Merredin in Western Australian. In this study, we thus investigated the effects of soil reflectance both in theory and in practice and were intended to raise more attention for utilization of soil reflectance when applying PROSAIL to retrieve LAI.

The background correction of reflectance via vegetation and background binary classification was to tackle the difference between modelling and observation. The soil reflectance used in PROSAIL assumes to be the reflectance of bare soil background [36, 40]. In fact, the background pixels might be a mixture of soil, plant residuals, and weeds instead of pure soil, which resulted in predicted LAI away from the expected value. A prior vegetation-background classification was proposed to be used to eliminate background effects [28], which was used to aid background correction to improve LAI prediction in our study (Figure 6; Figure S10). For the same RFR model (e.g., Mp3s2 or Mp3s3, the “RFR+LCB method” effectively corrected the overestimation issues from the “RFR method” for estimation of low LAI corresponding to sparse vegetation coverage (Figure 6). The success of “RFR+LCB method” somehow depends on the binary classification accuracy, raising requirements for classification algorithm and image spatial resolution (Figure S10; Table 3). From this aspect, the “RFR+LCB method” might be able to predict green LAI at late stages in theory, given heads and senescence leaves were correctly classified into background class. Previous analysis indicated that the background correction is needed for LAI prediction at early stage with sparse canopy. In this study, the NDVI threshold classification was chosen because the threshold of 0.5 was proved to effectively distinguish green leaves and soil when plants are small [4]. In theory, the mixed pixels will increase while pure soil pixels will decrease as image spatial resolution decreases, which hinders the accurate classification of vegetation and background [23] and therefore reduce the effectiveness of “RFR+LCB method.” This to some extent presents the applicability of UAV-based phenotyping with high spatial resolution in accurate prediction for low LAI.

### 4.2. Potential of the Method for Breeding

LAI can potentially aid genotype selection and adaptation assessment in breeding programs, and many indirect methods predicting LAI have been proposed [12, 28, 48]. One simple method is to calculate LAI from the fraction of intercepted photosynthetically active radiation (fIPAR) using Beer-Lambert Law with a constant extinction coefficient (*K*) estimated based on experience and prior observations. However, our study indicated that this method (i.e., “fIPAR” method) could not systematically achieve accurate estimation of LAI across growing stages for varying genotypes under varying treatments (Figure 6; Table 3). We observed a negative relationship between *k* and seasonal changes in LAI, i.e., *k* decreased with increasing LAI (Figure S9), consistent with previous findings summarized in a meta-analysis [37]. Using *K* estimates for each field experiment using the entire datasets (*K* = 0.81 for Exp16, *K* = 0.70 for Exp19) (assuming LAI was known) did improve LAI prediction (Figure S9b), which indicated that *K* was situation-specific. In fact, a more diverse *K* value was observed from our field experiments, varying from treatment to treatment in the range of 0.3~2.2 (Figure S9(c)).

Empirical models generally can achieve accurate LAI estimation in field conditions [22, 49] but might be unsuitable to make predictions in other conditions different to experimental conditions from which training data were collected [35]. For example, it was reported that for the same field experiment, predictive models built from mono-temporal VIs achieved accurate LAI estimation for wheat from UAV multispectral images for each phenotyping date (RRMSE=12~23%), but RRMSE increased to 20~40% and 17~35%, respectively, for models built from multi- and full-temporal VIs [50]. In contrast, the “RFR+LCB” synthetic-derived method robustly and accurately predicted LAI of wheat across growing stages (RRMSE=21~29%) and contrasting treatments (RRMSE=21~33%) (Table S2), which is useful for investigating LAI dynamics in breeding programs including a massive number of genotypes.

Both computationally intensive physical methods and more efficient hybrid methods are essentially able to accurately predict LAI under broad conditions [33, 34, 51]. However, these methods may fail to achieve accurate LAI estimation from spectral data due to rough spatial resolution from satellite images [13, 31], insufficient utilization of reflectance information related to VI selection [7], inappropriate algorithms used for building predictive model [14], or background and saturation effect [32]. Additionally, our study investigated an underestimation of LAI for LAI above 4 or 5 m^2^ m^−2^ depending on the condition analyzed (Figure 6, Figure S10), which was also reported in previous studies regardless of the methodologies used [32, 34]. This underestimation problem was documented as a saturation effect of reflectance and currently no effective methods have solved it [28]. RFR can provide more robust predictions and is less prone to overfitting due to its attributes (i.e., ensemble mechanism, sample, and attribute disturbance) [44], which is the main reason for choosing RFR to develop a predictive model in this hybrid method. In addition to RFR, there are other machine learning approaches (e.g., support vector machine, Gaussian process regression, neural network) that have been proposed to estimate LAI [14, 31, 34, 51]. These methods should be able to achieve competitive accuracy with RFR, given application of the three solutions proposed in this study, i.e., calibration of parameter range, soil reflectance, and image soil background.

The simulation of canopy reflectance with PROSAIL in this study applied the ellipsoidal distribution [52] to describe the leaf inclination distribution of canopy, so that it can be parameterized with the average leaf inclination angle (ALA). It assumes a uniform distribution in azimuth and at a constant zenith angle ranging from 20° (more planophile) to 70° (more erectophile), which covers most situations of possible leaf inclination distribution. Mathematical models in PROSAIL relate LAI and leaf inclination angle based on gap fraction theory [36, 48]. For canopies with leaves overlapping that do not simultaneously fulfil the two conditions (i. no space between adjacent plants along the same row; ii. no space between rows as seen from viewing angle), the estimation of LAI based on gap fraction will be underestimated [2]. For a sparse canopy with very low LAI<0.3 at tillering stage (no leaves overlapping), the leaf clumping should not affect LAI prediction. The leaves start to clump in elongation stage (i.e., can see gaps between rows but not between plants within rows) where the LAI may be underestimated for the canopy with obvious gaps between adjacent plants or rows. For fully extended canopy which can be treated as randomly distributed in azimuth, the underestimation of LAI is more due to the saturation of spectral information. In general, the clumping effect has larger influence for LAI estimation for canopies of forests or row crops with large row spacing [2, 48]. The RTM model (i.e., PROSAIL) used in this study does not consider the clumping effect. A more detailed description of 3D distribution of vegetation elements can better account for the clumping effect on maize canopies, while wheat was showing only marginal clumping effects [2, 53, 54].

In addition to accurate LAI estimations, the “RFR+LCB method” showed potential to correctly rank the different plots in regard to growing stages and treatments (genotypes, densities, and water-nitrogen managements). This is potentially useful in breeding programs where rapid but sensitive field phenotyping methods are needed to discriminate the seasonal, genotypic, and environmental differences [55, 56]. For the RFR model used in “RFR method” or “RFR+LCB method,” the only unknown factor (i.e., soil reflectance) can be obtained in advance and ranges of other input parameters could be determined based on prior knowledge, which means the RFR model can be built ahead of time. Even though the measured soil reflectance could not be obtained due to the lack of related equipment like ASD, our proposed soil calibration approach provided a generic way to simulate underlying soil reflectance based on the reflectance of bare soil pixels from UAV spectral images and default soil reflectance available in PROSAIL model (Figure S11). The simulated soil reflectance contributed to a relatively high-quality synthetic dataset used for the training of RFR models, which appeared to provide similarly accurate LAI prediction like that trained over synthetic datasets with measured soil reflectance (Figure S12). The “RFR+LCB method” therefore shows potentials to achieve in-season LAI estimation and to simulate LAI dynamics at early stages before LAI reaches its maximum (prior to head emergence).

## 5. Conclusions

This study evaluated the practical ability of predicting LAI from canopy reflectance captured in field conditions of the RFR models completely trained from synthetic data generated by PROSAIL. In addition to the capability of RFR itself to deal with prediction issues, the practical prediction accuracy of RFR model largely depends on the similarity between synthetic data (used for training model) and experimental data (used to evaluate model performance). On the base of ensuring that RFR model is suitable to predict LAI in broad conditions varying in genotypes and treatments across multiple growing stages, we investigated three solutions (i.e., calibration of parameter range, soil reflectance, and image soil background) to improve the LAI prediction accuracy of RFR model from the aspect of increasing data similarity. A wider variation in LAI extends model's application range, but it correspondingly increases the difficulty to make accurate estimation. The p2 range is suitable to be used to train models that are able to predict LAI in broad situations with LAI<7, while p3 range is recommended to be used in models that are designed to predict LAI under particular situations with LAI<5 (refer to Table 2 for definition of p2 and p3 ranges). Compared to narrowing parameter ranges, the utilization of local soil reflectance in synthetic data is more effective to improve RFR model's prediction accuracy. Optimizing synthetic data via calibration of parameter range and soil reflectance is a means of adjusting simulation towards observation, while calibration of image soil background is to try to adjust observation towards simulation to increase the data similarity. At early growing stage when plants are small and canopies are sparse (LAI < 0.5), the application of background-corrected reflectance map can effectively improve RFR model's prediction accuracy of LAI. However, the overestimation of LAI for LAI>5 due to the saturation of spectra information has not been effectively addressed in our study. In addition, the clumping effect is not considered in our research method framework, and our results did not show obvious underestimation of LAI during this stage. Overall, based on radiative transfer modelling and machine learning, we developed a prediction model that is able to accurately predict LAI from a single data source—UAB-based multispectral images—in field conditions, given appropriate calibration of parameter range, soil reflectance, and image soil background.

## Figures and Tables

**Table 1:** Crop characteristics when phenotyping occurred between plant emergence and flag leaf stage. Development stage and leaf area index (LAI) are given for dates when unmanned aerial vehicle (UAV) phenotyping occurred. In Exp19, LAI values correspond to measurements from quadrate harvests done at similar dates for the UAV phenotyping. In Exp16, LAI values were interpolated based on quadrate measurements from the whole growing season. The Min, Mean, and Max, respectively, represent the minimum, average, and maximum value of LAI for all selected plots within an experiment at a specific stage.

Experiment	UAV phenotyping date (DAS)	Development stage	LAI (m^2^ m^−2^)		
			Min	Mean	Max
Exp16	18	Tillering	0.1	0.18	0.26
Exp16	40	Start of stem elongation	0.5	1.39	2.32
Exp16	59	Flag leaf	1.12	2.69	5.12
Exp19	36	Start of stem elongation	0.81	1.97	3.98
Exp19	62	Flag leaf	1.58	3.61	6.8

Note: DAS: days after sowing.

**Table 2:** Input parameter sets of the PROSAIL model for the simulations (p1, p2, p3). Input parameters were either fixed or followed a uniform distribution from a range of values presented in the square brackets (minimum value on the left, and maximum value on the right). Input parameters include Ns, Cab, Car, Cant, Cbrown, Cm, Cw, ALA, LAI, *hspot*, *psoil*, *SZA*, *VZA*, and *RAA*. Compared with p1, parameter inputs of p2 for Cm, Cw, and LAI were limited to narrower ranges based on observed values from field experimental data. Compared with p2, the range of LAI values in p3 was further limited to LAI range of 0-5 m^2^ m^−2^. Changes of parameter range in p1, p2, and p3 were formatted in bold.

Parameters	Set#1 (p1)	Set#2 (p2)	Set#3 (p3)	Distribution
Leaf properties				
*Ns*	[1,2.5]	[1,2.5]	[1,2.5]	Uniform
*Cab*	[0,90]	[0,90]	[0,90]	Uniform
*Car*	[0,20]	[0,20]	[0,20]	Uniform
*Cant*	0	0	0	Fixed
*Cbrown*	0	0	0	Fixed
*Cm*	[0.001,0.02]	[0.001,0.01]	[0.001,0.01]	Uniform
*Cw*	[0.001,0.05]	[0.001,0.03]	[0.001,0.03]	Uniform
Canopy architecture				
*ALA*	[20,70]	[20,70]	[20,70]	Uniform
*LAI*	[0,8]	[0,7]	[0,5]	Uniform
*hspot*	[0.01,0.5]	[0.01,0.5]	[0.01,0.5]	Uniform
Soil adjustment factor				
*psoil*	1	1	1	Fixed
Solar-object-sensor observation geometry				
*SZA*	[20,70]	[20,70]	[20,70]	Uniform
*VZA*	0	0	0	Fixed
*RAA*	[-90,90]	[-90,90]	[-90,90]	Uniform
				

*Notes:* leaf mesophyll structure parameter (*Ns*, unitless), leaf chlorophyll content (*Cab*, *μ*g cm^−2^), leaf carotenoid content (*Car*, *μ*g cm^−2^), leaf anthocyanins content (*Cant*, *μ*g cm^−2^), leaf brown pigment (*Cbrown*, unitless), leaf water content or leaf equivalent water thickness (*Cw*, g cm^−2^), leaf dry matter content (*Cm*, g cm^−2^), average leaf inclination angle (*ALA*, degree), leaf area index (*LAI*, m^2^ m^−2^), hot-spot parameter (*hspot*, m m^−1^), soil adjustment factor (psoil, unitless), solar zenith angle (*SZA*, degree), viewing zenith angle (*VZA*, degree), relative azimuth angle (*RAA*, degree).

**Table 3:** Estimation accuracy (*r*, *R*^2^, RMSE, RRMSE) of LAI predicted with three studied methods for two experiments (Exp16, Exp19) for the full range of LAI or only LAI <=5. The three methods are “fIPAR method,” using Beer-Lambert to predict LAI from the fraction of vegetation coverage with setting *K* to a constant (0.5); “RFR method,” using random forest regression models trained on synthetic dataset varying in parameter range (p1, p2, p3) and soils (s1, s2, s3) to predict LAI from original reflectance; “RFR+LCB method,” using the same models to predict LAI from background-corrected reflectance. “(LAI≤5)” denotes only experimental data limited to 5 m^2^ m^−2^ were used.

Experimental data	Method	Model	*r*	*R* ^2^	RMSE (m^2^ m^−2^)	RRMSE (%)
Exp16 (*n* = 252)	fIPAR	/	0.95	0.91	0.43	30
	RFR	Mp1s1	0.79	0.63	1.51	106
	RFR+LCB	Mp1s1	0.90	0.81	0.60	42
	RFR	Mp1s2 (Mp1s2∗)	0.92 (0.92)	0.85 (0.85)	0.54 (0.56)	38 (39)
	RFR+LCB	Mp1s2 (Mp1s2∗)	0.94 (0.93)	0.89 (0.87)	0.45 (0.55)	31 (39)
	RFR	Mp2s1	0.75	0.56	1.47	104
	RFR+LCB	Mp2s1	0.90	0.80	0.60	42
	RFR	Mp2s2 (Mp2s2∗)	0.94 (0.94)	0.89 (0.89)	0.46 (0.40)	33 (28)
	RFR+LCB	Mp2s2 (Mp2s2∗)	0.95 (0.95)	0.91 (0.90)	0.36 (0.40)	25 (28)
Exp19 (*n* =144)	fIPAR	/	0.83	0.69	1.13	40
	RFR	Mp1s1	0.43	0.18	1.58	57
	RFR+LCB	Mp1s1	0.54	0.29	1.31	47
	RFR	Mp1s3 (Mp1s3∗)	0.80 (0.81)	0.63 (0.65)	0.93 (1.04)	33 (37)
	RFR+LCB	Mp1s3 (Mp1s3∗)	0.78 (0.80)	0.61 (0.64)	0.93 (1.05)	33 (38)
	RFR	Mp2s1	0.64	0.41	1.17	42
	RFR+LCB	Mp2s1	0.67	0.46	1.03	37
	RFR	Mp2s3 (Mp2s3∗)	0.80 (0.84)	0.64 (0.70)	0.84 (0.81)	30 (29)
	RFR+LCB	Mp2s3 (Mp2s3∗)	0.80 (0.83)	0.63 (0.69)	0.86 (0.84)	31 (30)
Exp16 (LAI<=5) (*n* = 251)	fIPAR	/	0.96	0.91	0.42	30
	RFR	Mp3s2 (Mp3s2∗)	0.95 (0.95)	0.91 (0.91)	0.47 (0.41)	33 (29)
	RFR+LCB	Mp3s2 (Mp3s2∗)	0.96 (0.96)	0.91 (0.91)	0.37 (0.41)	26 (29)
Exp19 (LAI<=5) (*n* =130)	fIPAR	/	0.78	0.60	1.05	43
	RFR	Mp3s3 (Mp3s3∗)	0.80 (0.82)	0.64 (0.67)	0.61 (0.64)	25 (26)
	RFR+LCB	Mp3s3 (Mp3s3∗)	0.79 (0.82)	0.62 (0.67)	0.64 (0.67)	26 (27)

## Data Availability

The detailed protocols and analysis results can be found in supplementary materials. Details and source code of the PROSAIL model used in this study are openly available at http://teledetection.ipgp.jussieu.fr/prosail/. Other data and source code supporting this work are available from the corresponding author upon request.

## Supplementary Materials

Supplementary Materials Method S1: Interpolation of LAI. Figure S1: Growth curve of leaf area index in 2016. Method S2: Vegetation-background binary classification and background correction. Method S3: Calculation of band reflectance. Figure S2: Spectral response coefficient for each band of MicaSense RedEdge camera. Figure S3: Locations of the two experiments in Gatton campus, and plots with LAI measurements. Figure S4: Reflectance of soil under dry and wet conditions. Figure S5: Difference of simulated canopy reflectance caused by Cm and Cw. Figure S6: Simulated canopy reflectance spectra with PROSAIL for various LAI. Figure S7: Evaluation of RFR models for different levels of predicted LAI. Figure S8: Known LAI against predicted LAI or predicted residual. Figure S9: Observed LAI against fIPAR or predicted LAI as well as extinction coefficient. Figure S10: Observed LAI against LAI predicted with RFR-based methods. Figure S11: Calibration of soil characteristics. Figure S12: Observed LAI against LAI predicted with different soil reflectance. Table S1: RFR models and their corresponding training and test datasets. Table S2: Segmentation analysis for LAI estimation accuracy based on “RFR+LCB method”. Table S3: Paired sample *t*-test of observed LAI between different levels.
