# Supplementary material for: Unsupervised Plot-Scale LAI Phenotyping via UAV-Based Imaging, Modelling, and Machine Learning
Source: Plant Phenomics. 2022 Jul 2;2022:9768253. doi: 10.34133/2022/9768253 (PMC9317541; doi:10.34133/2022/9768253)
Supplement: Supplementary Materials — Method S1: Interpolation of LAI. Figure S1: Growth curve of leaf area index in 2016. Method S2: Vegetation-background binary classification and background correction. Method S3: Calculation of band reflectance. Figure S2: Spectral response coefficient for each band of MicaSense RedEdge camera. Figure S3: Locations of the two experiments in Gatton campus, and plots with LAI measurements. Figure S4: Reflectance of soil under dry and wet conditions. Figure S5: Difference of simulated canopy reflectance caused by Cm and Cw. Figure S6: Simulated canopy reflectance spectra with PROSAIL for various LAI. Figure S7: Evaluation of RFR models for different levels of predicted LAI. Figure S8: Known LAI against predicted LAI or predicted residual. Figure S9: Observed LAI against fIPAR or predicted LAI as well as extinction coefficient. Figure S10: Observed LAI against LAI predicted with RFR-based methods. Figure S11: Calibration of soil characteristics. Figure S12: Observed LAI against LAI predicted with different soil reflectance. Table S1: RFR models and their corresponding training and test datasets. Table S2: Segmentation analysis for LAI estimation accuracy based on “RFR+LCB method”. Table S3: Paired sample t-test of observed LAI between different levels. [file 9768253.f1.docx]

Unsupervised plot-scale LAI phenotyping via UAV-based imaging, modelling and machine learning

Qiaomin Chen ^1, 2 *^, Bangyou Zheng ^2^, Karine Chenu ^3^, Pengcheng Hu ^1, 2^, Scott C. Chapman ^1 *^

**Supplemental materials**

# Method S1: Interpolation of LAI

The missing LAI on phenotyping dates in 2016 were interpolated with a fitted piecewise function: using a logistic function and beta function before and after LAI reaching its maxima, respectively (see Eq. (1)).

| $LAI=\left\{ \begin{aligned} \begin{matrix} c+\frac{d-c}{1+exp(b(ATT-e))} & ATT\leq argmax(LAI) \end{matrix} \\ \begin{matrix} \frac{1}{B\left( P,Q \right)}{ATT}^{P-1}{(1-ATT)}^{Q-1} & ATT>argmax(LAI) \end{matrix} \end{aligned} \right.$ | (1) |
| --- | --- |

where ATT (0<ATT<1) represents the standardized value of accumulative thermal time from emergence, calculated by summing the daily effective thermal time (TT, °C day). TT is set to 0 when daily mean temperature is lower than 0 or higher than 37, otherwise, it is equal to the value of daily mean temperature; argmax(LAI) is the ATT value generating the maximum LAI; c is the lower asymptote of LAI (in this study, it is set to zero, c=0); d is the higher asymptote of LAI; e is the value of ATT producing a LAI halfway between c and d; b is the slope around the inflection point; P and Q are the two parameters controlling the shape of curve (P>0, Q>0); B(P,Q) is known as the “beta function”. The fitted growth curves of LAI during growing season were presented in Fig. S1.


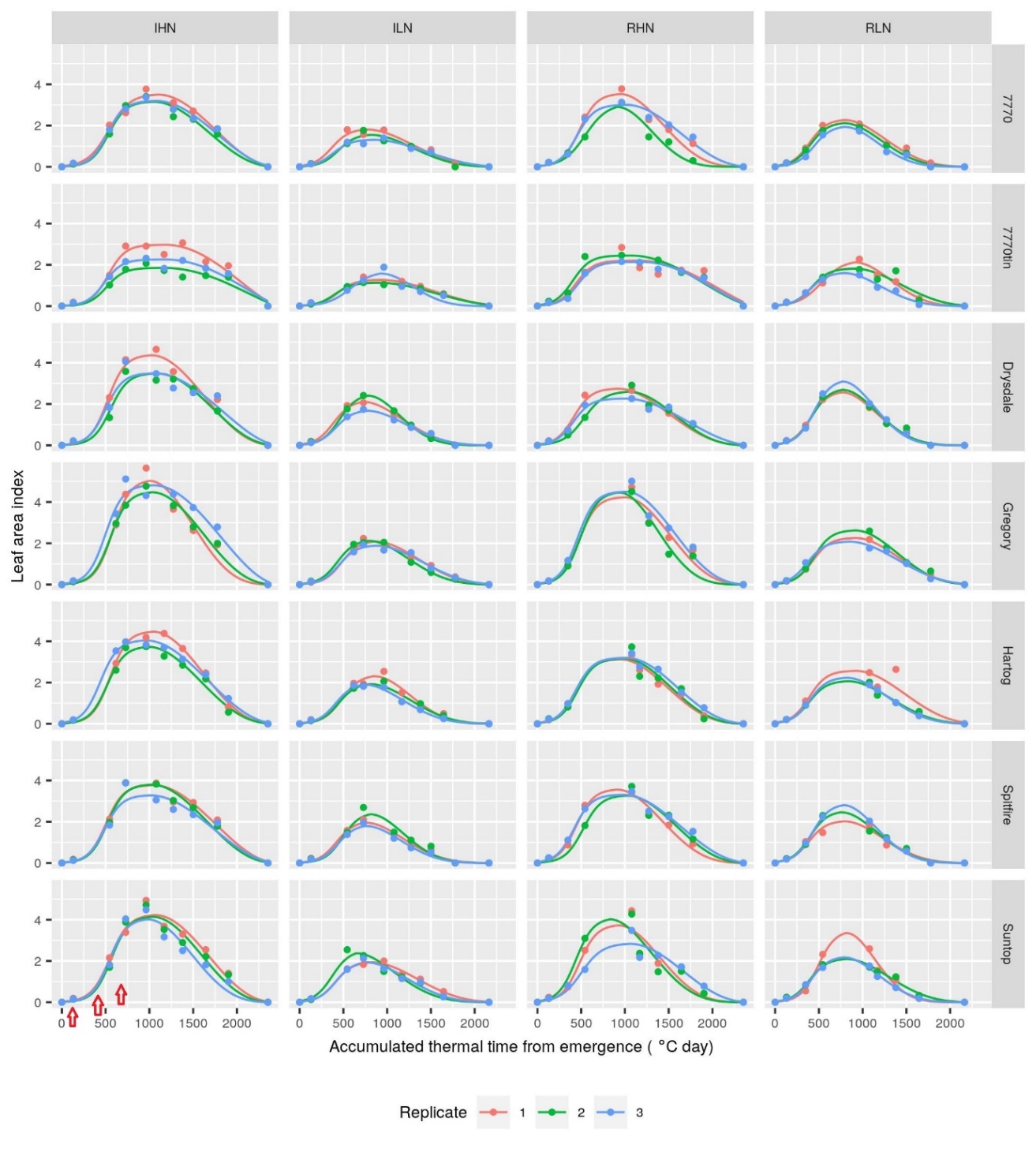


Fig. S1 Growth curve of leaf area index in 2016. IHN represents irrigated and high nitrogen treatment; ILN represents irrigated and low nitrogen; RHN represents rainfed and high nitrogen; RLN represents rainfed and low nitrogen. The red arrows denote the three phenotyping dates at wheat early stage from plant emergence to flag leaf.

# Method S2: Vegetation-background binary classification and background correction

**Vegetation-background binary classification**: This vegetation-background binary classification is based on NDVI threshold (NDVI_soil_). Two reflectance bands (NIR and Red) are used to calculate the NDVI map based on the formula: NDVI=(NIR-Red)/(NIR+Red). If the NDVI value of the pixel is less than or equal to the threshold (NDVI ≤ NDVI_soil_), this pixel will be classified as background; otherwise, if NDVI > NDVI_soil_, this pixel will be classified as vegetation. Based on this threshold, the NDVI map can be converted into a vegetation-background binary map. The determination of the NDVI threshold is realized through a two-step procedure. Step 1, twelve plots evenly distributed in the trial were randomly selected, and the vegetation and background regions within each plot were identified from the NDVI map via visual inspection; then, the fcover (i.e., visually-inspected fcover) was calculated by dividing the vegetation area from the plot area for each plot. Step 2, calculate the fcover (i.e., threshold-derived fcover) for selected plots by iteratively adjusting NDVI threshold at fixed interval (i.e., 0.05) in the range of 0.05-0.8, so that the optimal NDVI_soil_ for the corresponding plot can be determined by minimizing the objective function below:

| ${NDVI}_{soil}=argmin(\sum_{j=1}^{m} \sum_{i=1}^{n} \left( f_{c,i}-f_{t,i,j} \right)^{2})$ | (2) |
| --- | --- |

where n represents the number of selected plots (here n=10); m represents the number of NDVI threshold used in the iterative process (here m=16); f_v,i_ represents the visually-inspected fcover of the i^th^ selected plot; f_t,i,j_ represents the threshold-derived fcover of the i^th^ selected plot when NDVI_soil_ is set to the j^th^ threshold in the iterative optimization. The NDVI threshold was determined with this procedure for each phenotyping date, respectively, which resulted in the three thresholds used in this study (i.e., 0.5 for tillering stage, 0.65 for stem elongation stage and 0.75 for flag leaf stage).

**Background correction**: Once the vegetation-background binary classification map is generated, it can be used as a mask to locate the position of background pixels in each reflectance map, as the classification map and the reflectance map is completely aligned. The default soil reflectance (s1), the measured soil reflectance (s2 and s3) and the simulated soil reflectance (s2* and s3*) in the range of 400-2500 nm at 1nm interval were resampled into band reflectance based on spectral response coefficient as demonstrated in Method S3. For each band, the value of background pixels of original reflectance map was replaced with the band value of corresponding soil reflectance used in the specific synthetic dataset, which resulted in the “background-corrected” reflectance map. Taking Fig. 3 as example, if the RFR model used to predict LAI was trained over synthetic data in which the measured soil reflectance for Exp16 (s2) was used, then the value of all background pixels of the original reflectance map for blue band was replaced with the blue band value of s2. Likewise, if the RFR model was trained over synthetic data with the simulated soil reflectance for Exp16 (s2*), then the value of all background pixels of the original reflectance map for blue band was replaced with the blue band value of s2*. This correction was only applied on background pixels and did not change the values of vegetation pixels.

# Method S3: Calculation of band reflectance

The simulated band reflectance for each band of MicaSense RedEdge camera is resampled from hyperspectral reflectance (1 nm interval) simulated by PROSAIL based on spectral response coefficient provided by MicaSense (Fig. S2). The band reflectance can be calculated as follows:

| $\rho_{i}=\frac{\sum_{j=\lambda_{i,min}}^{\lambda_{i,max}} \varphi_{i,j}\times\rho_{j}}{\sum_{j=\lambda_{i,min}}^{\lambda_{i,max}} \varphi_{i,j}}$ | (3) |
| --- | --- |

where $\rho_{i}$ represents simulated band reflectance for i^th^ band of the camera; $\rho_{j}$ represent the simulated hyperspectral reflectance for j^th^ wavelength; $\varphi_{i,j}$ represents the response coefficient of the i^th^ band of the camera for the j^th^ wavelength; $\lambda_{i,min}$ and $\lambda_{i,max}$ represent the lower- and upper-band wavelength limits for the i^th^ band, respectively.


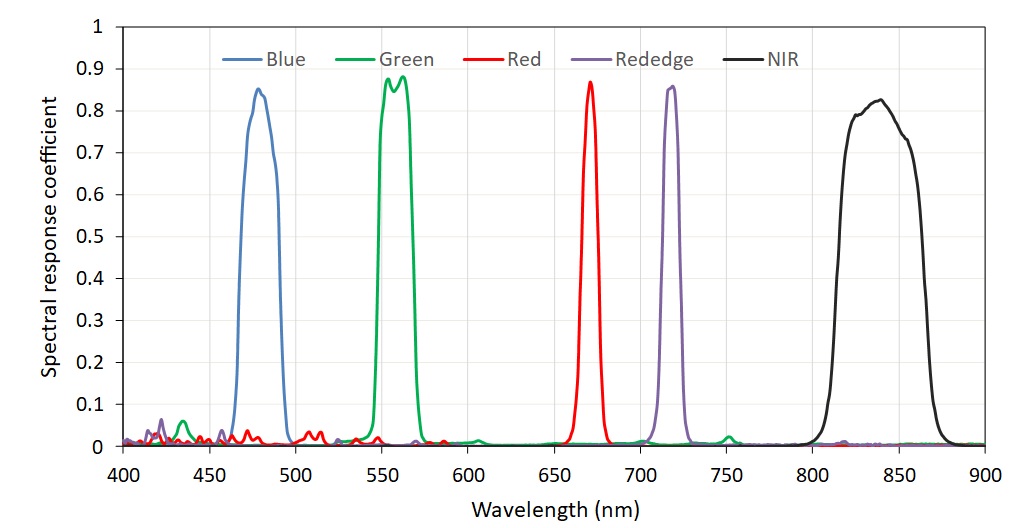


Fig. S2 Spectral response coefficient for each band of Micasense RedEdge camera. The centre wavelength and bandwidth for each band is as: Blue (475 nm center wavelength, 20 nm bandwidth), Green (560 nm, 20 nm), Red (668 nm, 10 nm), Rededge (717 nm, 10 nm), and Near Infrared (NIR: 840 nm, 40 nm)

# Supplemental Figures


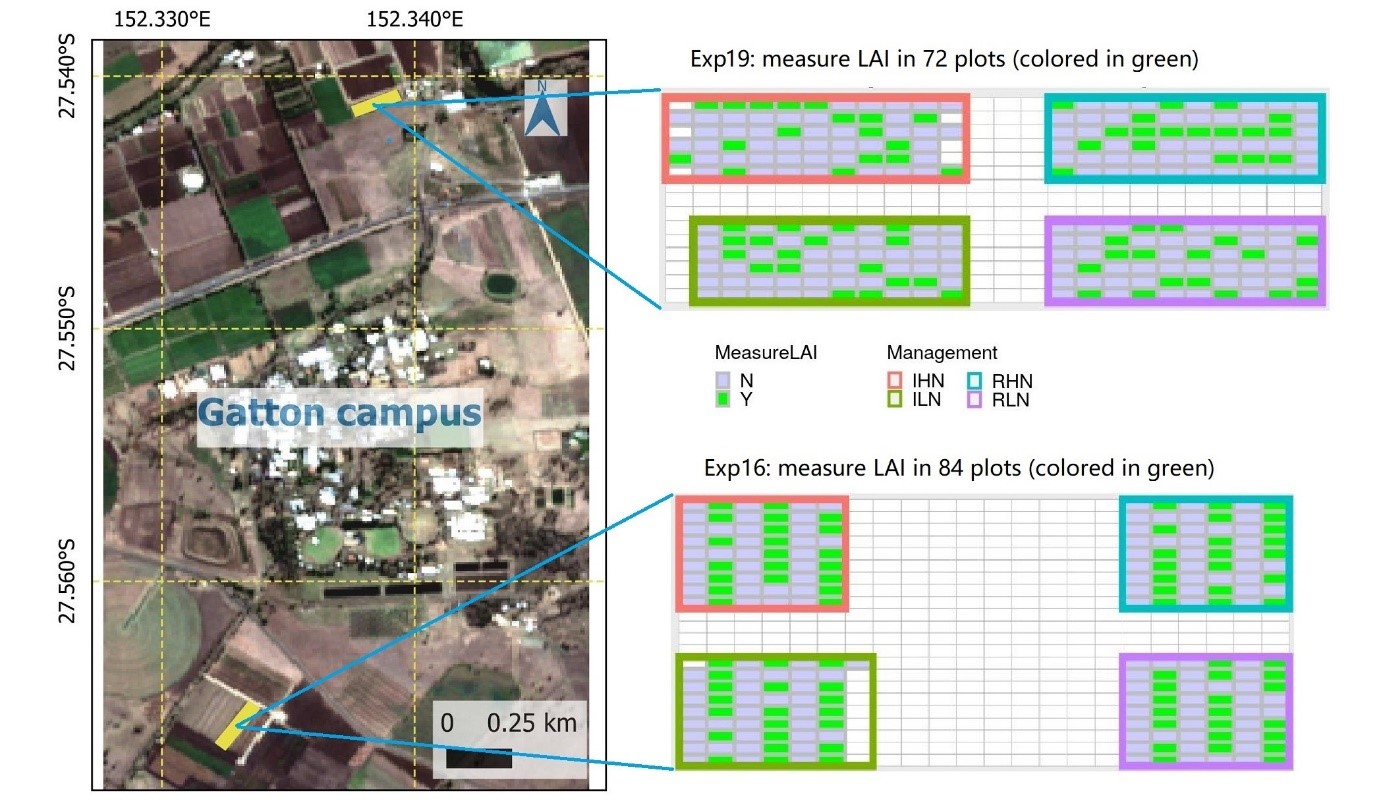


Fig. S3 Locations of the two experiments in Gatton campus, and plots with LAI measurements. The four management blocks denote different water-nitrogen combinations: irrigated and high nitrogen (IHN), irrigated and low nitrogen (ILN), rainfed and high nitrogen (RHN), rainfed and low nitrogen (RLN). Plots colored in green in the four blocks represent those selected for manually measuring leaf area index (LAI). Exp16 and Exp19 represent wheat trials conducted in 2016 and 2019, respectively.


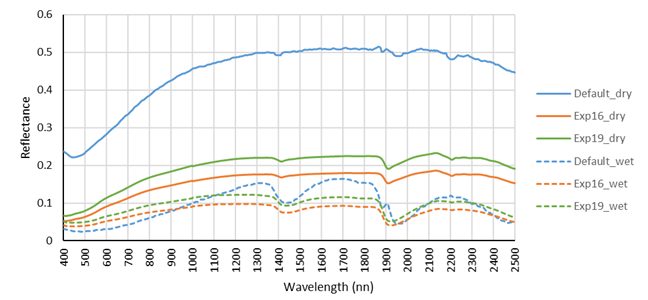


Fig. S4 Reflectance of soil under dry and wet conditions extracted from the PROSAIL model and measured in field experiments. The Default_dry and Default_wet represent the reflectance of default soil used in the model under dry and wet conditions, respectively. Likewise, Exp16_dry and Exp16_wet represent measured soil reflectance of Exp16, while Exp19_dry and Exp16_wet represent measured soil reflectance of Exp19.


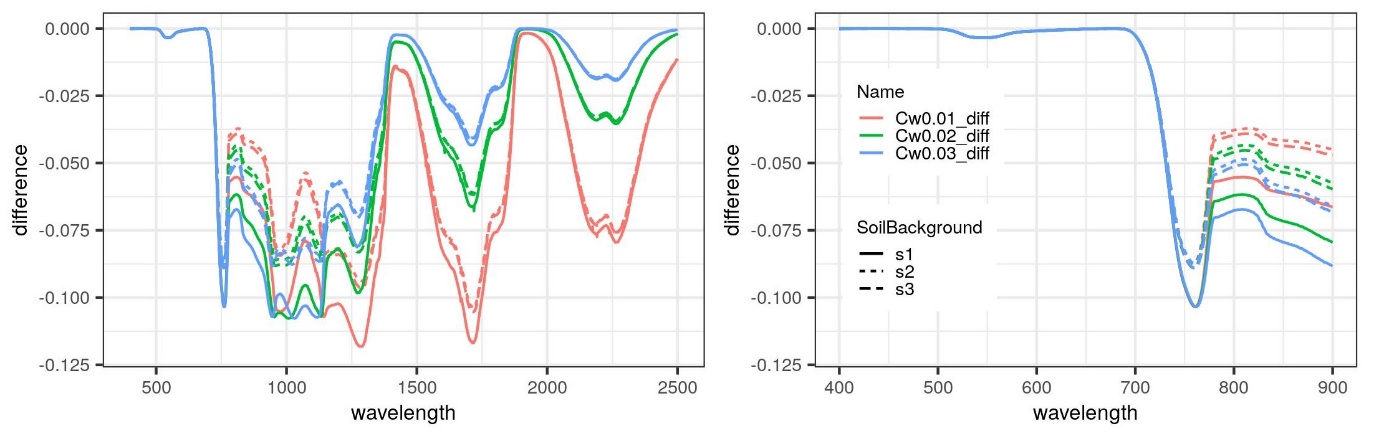


Fig. S5 Difference of simulated canopy reflectance with PROSAIL caused by changing Cm from 0.001 to 0.01 at specific Cw level and soil reflectance. Cw was set at three levels (i.e., 0.01, 0.02, 0.03) and other parameters in the PROSAIL model were set at the average values of p2 (i.e., Ns=1.75, Cab=45, Car=10, Cant=0, Cbrown=0, ALA=45, LAI=3.5, hspot=0.255, psoil=1, SZA=45, VZA=0, RAA=0) in Table 2. The three soils corresponded to the default soil of PROSAIL (s1) and measured soil of Exp16 (s2) and Exp19 (s3).


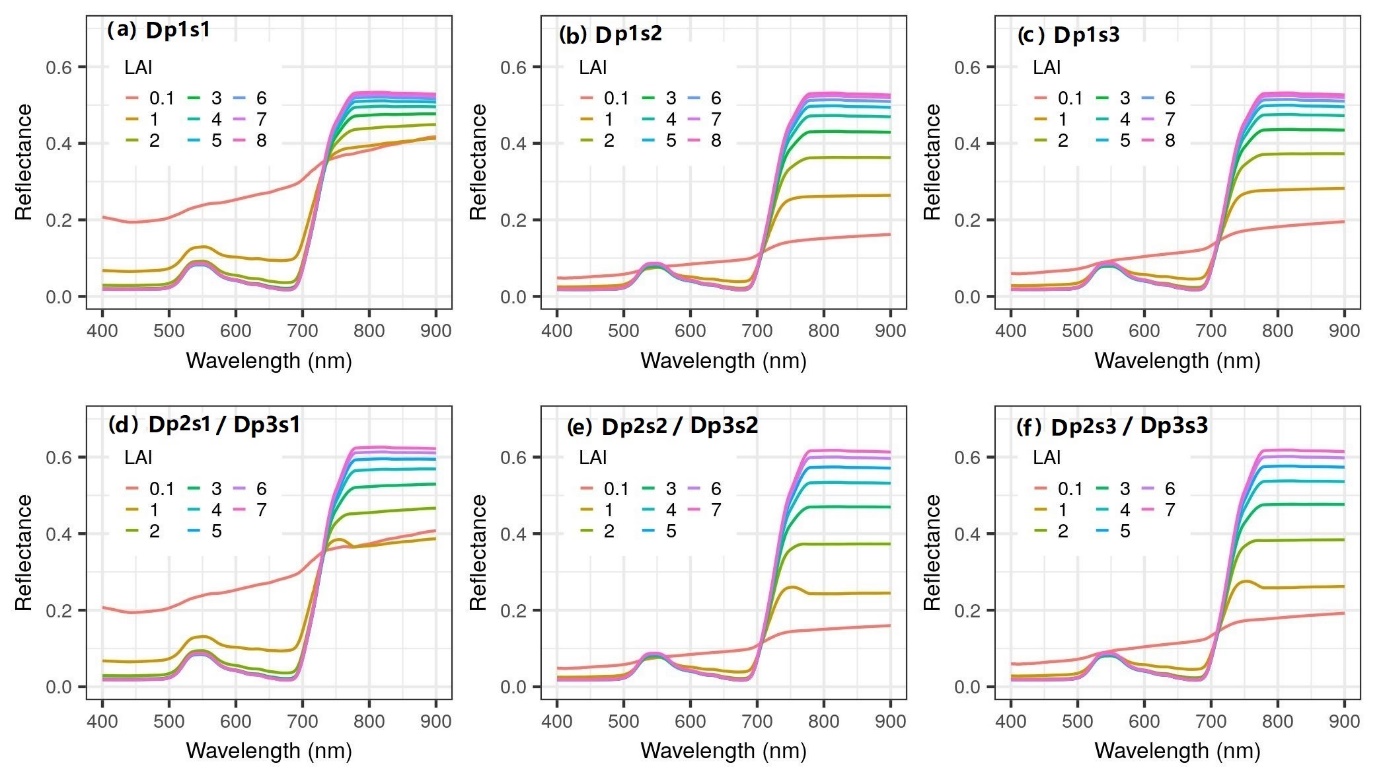


Fig. S6 The simulated canopy reflectance spectra with PROSAIL for various LAI using different parameter ranges and soil reflectance. Other parameters in the PROSAIL model were set at the average of values in Table 2 (i.e., Ns=1.75, Cab=45, Car=10, Cant=0, Cbrown=0, Cm=0.0105, Cw=0.0255, ALA=45, hspot=0.255, psoil=1, SZA=45, VZA=0, RAA=0 for p1). Dataset name consisted of two parts: parameter range and soil background; for example, “Dp1s1” denotes a dataset generated with p1 and model soil reflectance. s1, s2 and s3 represents the default soil of the PROSAIL model and measured soil of Exp16 and Exp19, respectively. p2 and p3 have the same simulated canopy reflectance for LAI <=5 as values for all other parameters were the same.


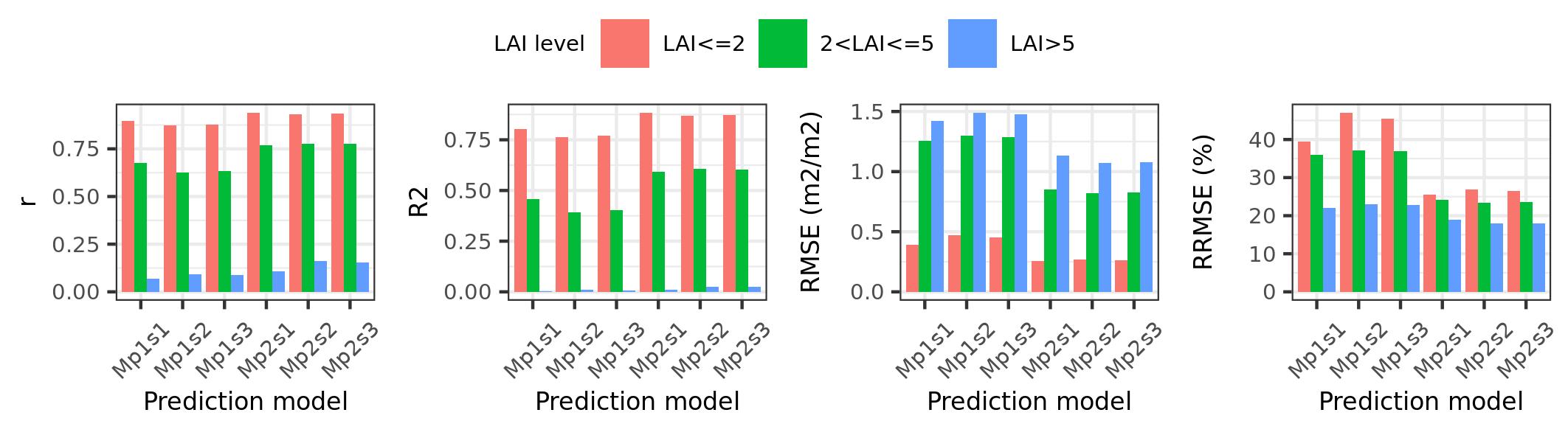


Fig. S7 Evaluation of RFR models for different levels of predicted LAI in the studied synthetic datasets. Values of r, R2, RMSE and RRMSE were calculated between known and predicted LAI for test datasets for each model trained on synthetic dataset varying in parameter range and soil (Table 2). Known LAI correspond to input LAI values used to run PROSAIL.


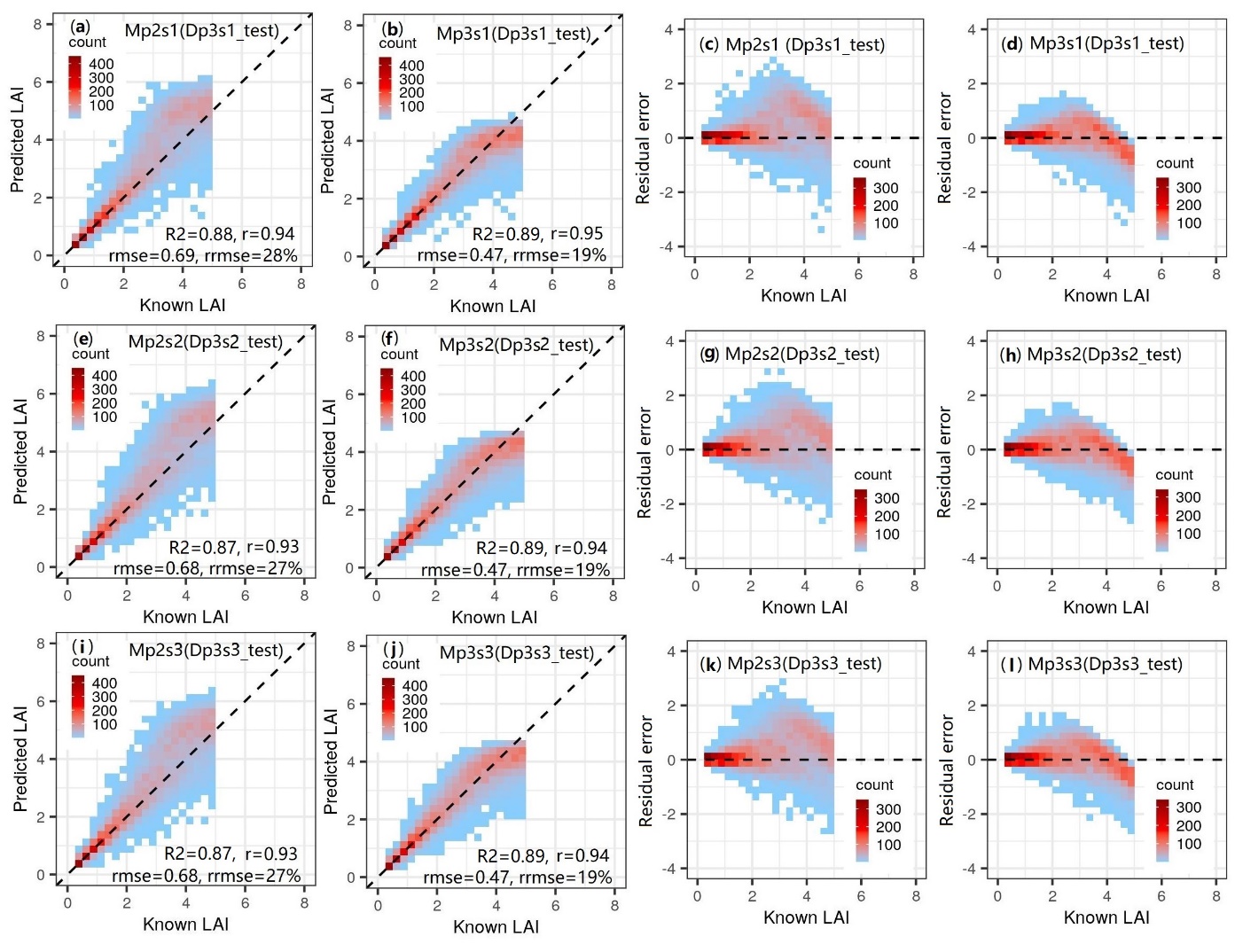


Fig. S8 Known LAI against predicted LAI (a, b, e, f, I, j) or predicted residual (c, d, g, h, k, l). LAI was predicted with RFR models trained on synthetic data produced by PROSAIL for parameter range p2 (0<LAI≤7) or p3 (0<LAI≤5) and soil type (s1, s2, s3). All models (p2 or p3) were tested on synthetic data generated from p3 parameter range (0<LAI≤5). Known LAI correspond to input LAI values used to run PROSAIL. Residual error corresponds to the difference of known LAI subtracted from predicted LAI.


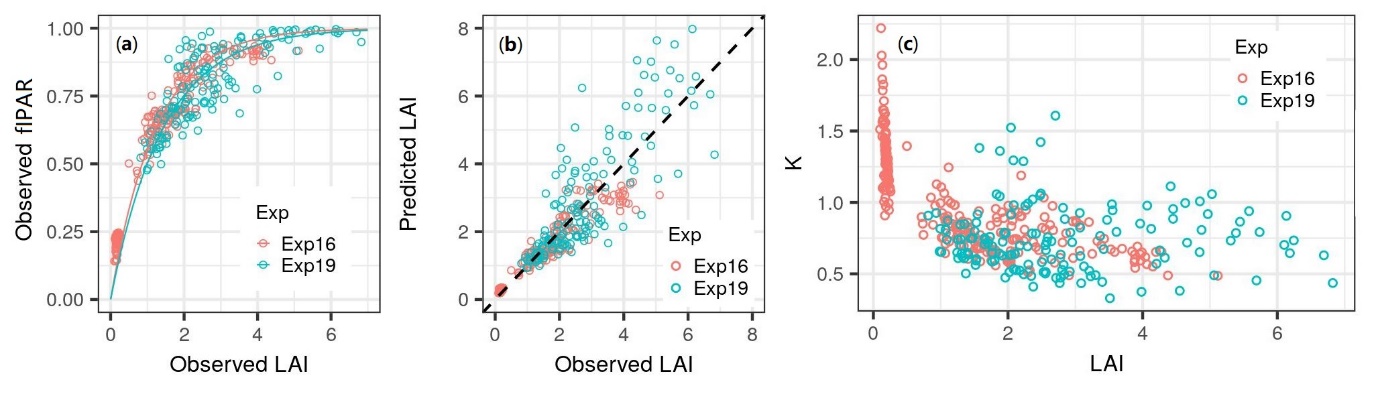


Fig. S9 Observed LAI against the fraction of intercepted photosynthetically active radiation (fIPAR) (a) or predicted LAI (b), and instant extinction coefficient (K). (a) The solid line was the fitted curve for each experiment based on Beer-Lambert Law, i.e., fIPAR=1−exp(−K×LAI), with fitted K value of 0.81 for Exp16 and 0.70 for Exp19. (b) The values of predicted LAI were calculated with “fIPAR” method, i.e., computed from fIPAR and K based on LAI = −ln(1−fIPAR)/K, where K was set to the fitted value of fitted curve in (a). (c) The values of K were calculated with averaged value of measured fIPAR and observed LAI at plot level. Exp16 had 252 data points and Exp19 had 144 data points.


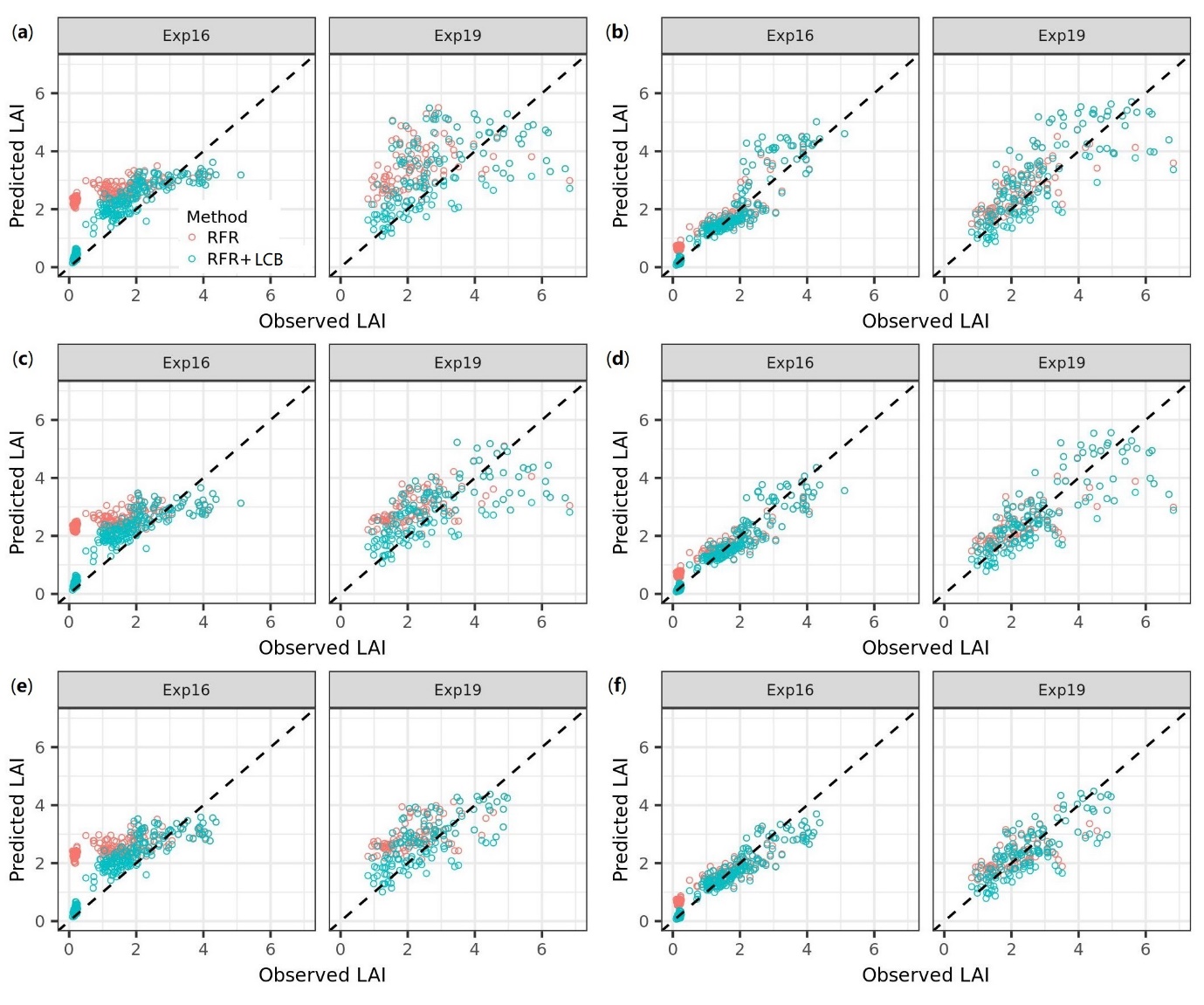


Fig. S10 Observed LAI against LAI predicted with RFR models trained with synthetic datasets varying in soil background (s1, default soil: a, c, e; s2 or s3, local soil: b, d, f) and parameter sets (p1: a, b; p2: c, d; p3: e, f). Models trained with p1/p2 datasets were used to predict LAI up to 7 m^2^ m^-2^ while models trained with p3 datasets were used to predict LAI up to 5 m^2^ m^-2^. Models used in a specific subfigure were the same, but red symbols represent results predicted with “RFR” method while blue symbols predicted with “RFR+LCB” method.


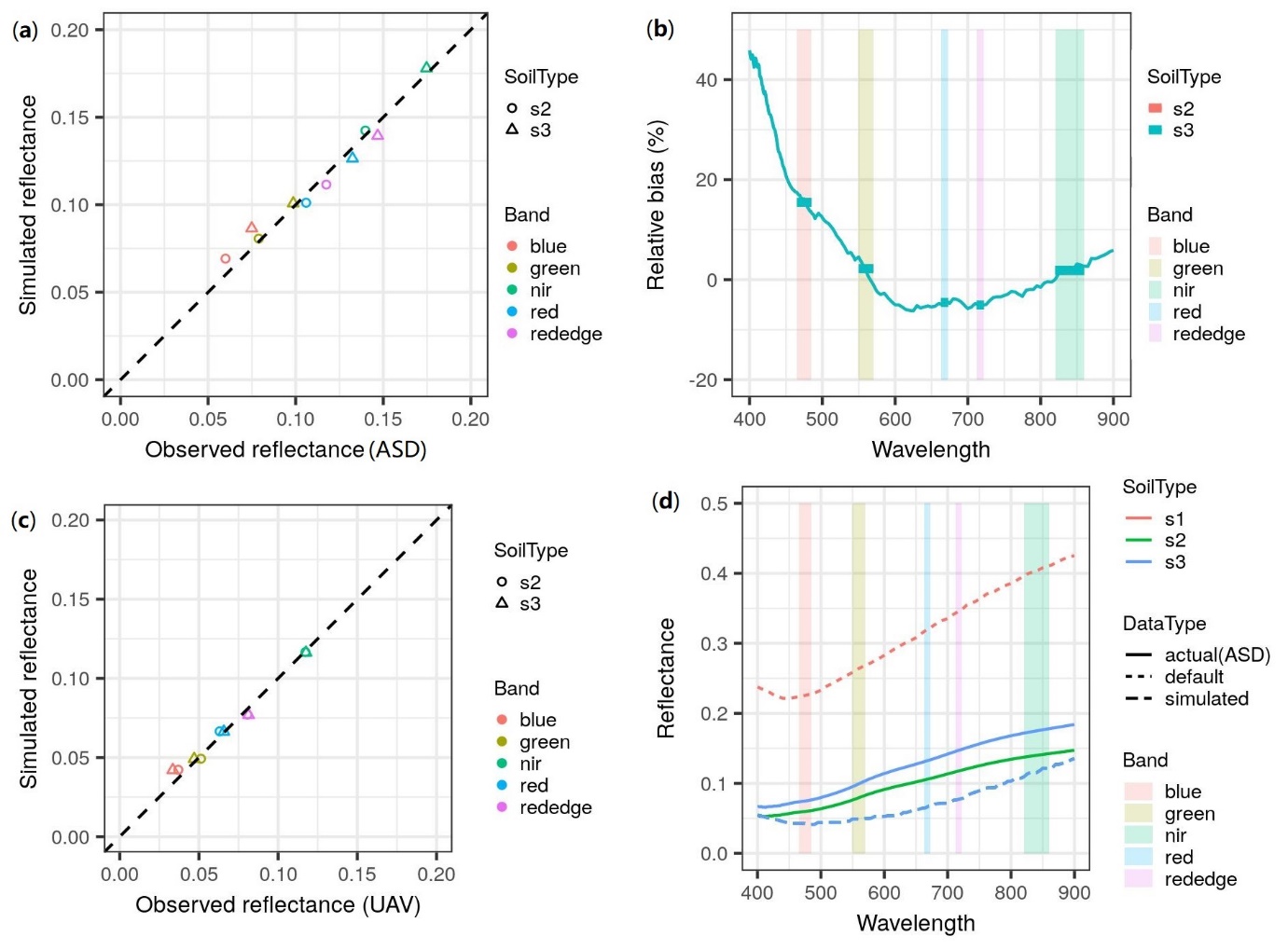


Fig. S11 Calibration of soil characteristics. (a) Resampled soil reflectance measured with ASD against its simulation generated with soil calibration approach. (b) Relative bias of simulated reflectance to measured reflectance for every wavelength and each band. (c) Soil reflectance retrieved from UAV multi-spectral images against its simulation generated with soil calibration approach. (d) Default soil reflectance (s1, dry condition) in PROSAIL model, measured (actual_ASD, dry condition) and simulated soil reflectance of two experiments (s2, s3). The simulated soil reflectance covered the range of 400-2500nm with 1-nm interval but only the results in 400-900 nm were presented here.


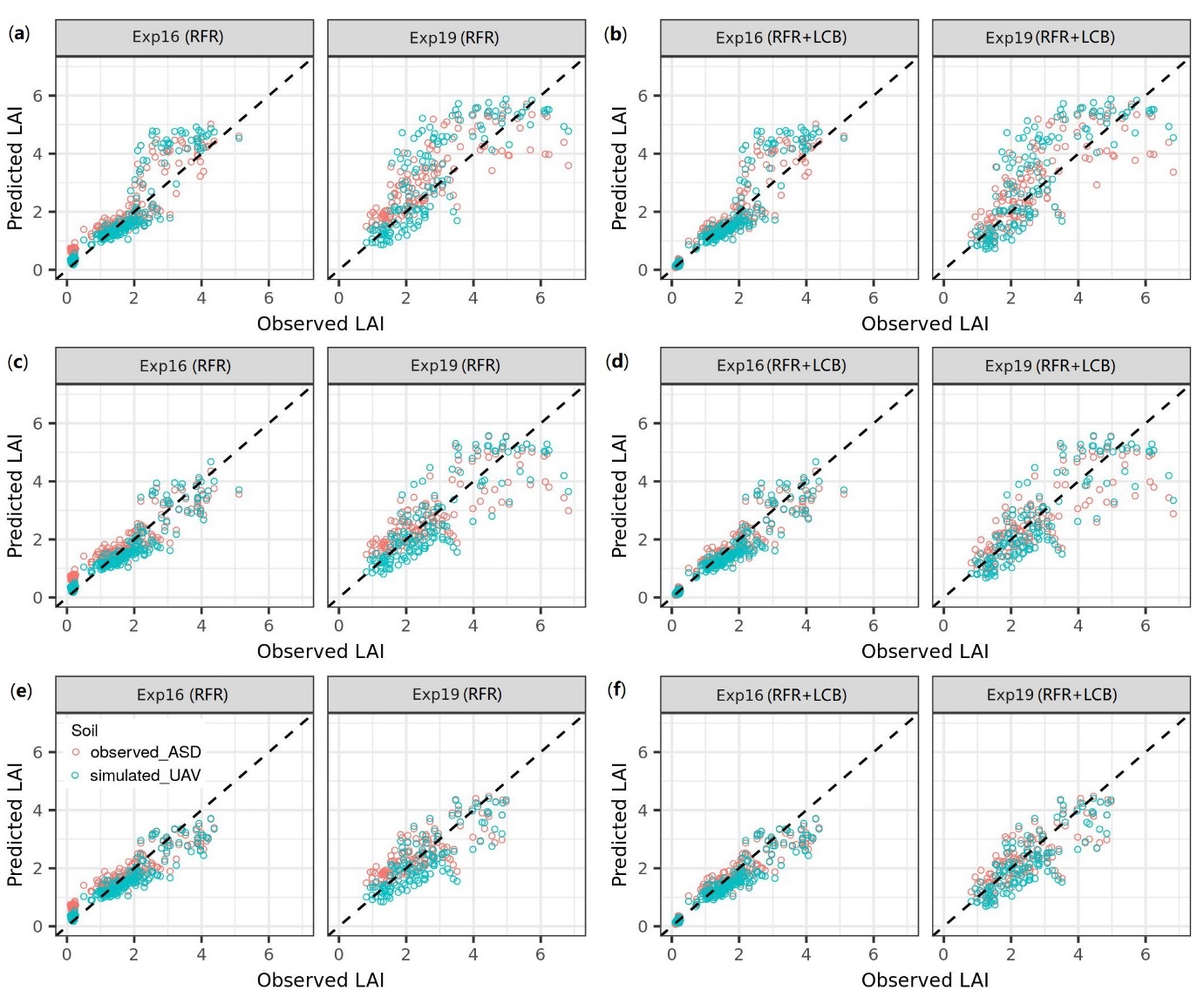


Fig. S12 Observed LAI against LAI predicted with RFR models trained with synthetic datasets varying in soil reflectance and parameter sets (p1: a, b; p2: c, d; p3: e, f). Models trained with p1/p2 datasets were used to predict LAI up to 7 m^2^ m^-2^ while models trained with p3 datasets were used to predict LAI up to 5 m^2^ m^-2^. The soil reflectance used in models in the same subfigure represented the same soil; however, red symbols represented soil reflectance measured with ASD (s2, s3), while blue symbols represented soil reflectance calibrated from UAV images (s2*, s3*).

# Supplemental Tables

Table S1 Random forest regression (RFR) models and their corresponding training and test datasets. All dataset starting with 'D’ were simulated with PROSAIL. Dataset with “(LAI I≤5)” denotes only experimental data limited to 5 m^2^ m^-2^ were used.

| Model | Training dataset | Test dataset (theoretical) | Test dataset (practical) |
| --- | --- | --- | --- |
| Mp1s1 | Dp1s1_training | Dp1s1_test, Dp1s2_test, Dp1s3_test | Exp16, Exp19 |
| Mp1s2 | Dp1s2_training | Dp1s2_test | Exp16 |
| Mp1s3 | Dp1s3_training | Dp1s3_test | Exp19 |
| Mp2s1 | Dp2s1_training | Dp2s1_test | Exp16, Exp19 |
| Mp2s2 | Dp2s2_training | Dp2s2_test, Dp3s2_test | Exp16, Exp16 (LAI≤5) |
| Mp2s3 | Dp2s3_training | Dp2s3_test, Dp3s3_test | Exp19, Exp19 (LAI≤5) |
| Mp3s1 | Dp3s1_training | Dp3s1_test | Exp16 (LAI≤5), Exp19 (LAI≤5) |
| Mp3s2 | Dp3s2_training | Dp3s2_test | Exp16 (LAI≤5) |
| Mp3s3 | Dp3s3_training | Dp3s3_test | Exp19 (LAI≤5) |
| Mp1s2* | Dp1s2*_training | / | Exp16 |
| Mp1s3* | Dp1s3*_training | / | Exp19 |
| Mp2s2* | Dp2s2*_training | / | Exp16 |
| Mp2s3* | Dp2s3*_training | / | Exp19 |
| Mp3s2* | Dp3s2*_training | / | Exp16 (LAI≤5) |
| Mp3s3* | Dp3s3*_training | / | Exp19 (LAI≤5) |

Table S2 Estimation accuracy of LAI up to 5 m2 m-2 for different levels of four groups (i.e., growing stage characterized by days after sowing (DAS), genotype, density and water-nitrogen management) for two experiments. Model Mp3s2 and Mp3s3 were used in “RFR+LCB method” to predict LAI for Exp16 and Exp19, respectively.

| Experiment data | Group | Level | Model | r | R^2^ | RMSE (m^2^ m^-2^) | RRMSE (%) | Sample number |
| --- | --- | --- | --- | --- | --- | --- | --- | --- |
| Exp16 | DAS | 18 | Mp3s2 | 0.58 | 0.34 | 0.05 | 29 | 84 |
| Exp16 | DAS | 40 | Mp3s2 | 0.67 | 0.45 | 0.28 | 20 | 84 |
| Exp16 | DAS | 59 | Mp3s2 | 0.82 | 0.66 | 0.57 | 21 | 83 |
| Exp19 | DAS | 36 | Mp3s3 | 0.69 | 0.47 | 0.55 | 28 | 72 |
| Exp19 | DAS | 62 | Mp3s3 | 0.69 | 0.47 | 0.73 | 24 | 58 |
| Exp16 | Genotype | Spitfire | Mp3s2 | 0.96 | 0.93 | 0.40 | 26 | 36 |
| Exp16 | Genotype | 7770 | Mp3s2 | 0.96 | 0.93 | 0.27 | 21 | 36 |
| Exp16 | Genotype | 7770tin | Mp3s2 | 0.96 | 0.93 | 0.21 | 21 | 36 |
| Exp16 | Genotype | Suntop | Mp3s2 | 0.97 | 0.93 | 0.37 | 24 | 36 |
| Exp16 | Genotype | Gregory | Mp3s2 | 0.97 | 0.95 | 0.39 | 25 | 36 |
| Exp16 | Genotype | Drysdale | Mp3s2 | 0.93 | 0.86 | 0.48 | 33 | 36 |
| Exp16 | Genotype | Hartog | Mp3s2 | 0.97 | 0.94 | 0.38 | 25 | 35 |
| Exp19 | Genotype | Beaufort | Mp3s3 | 0.71 | 0.51 | 0.75 | 33 | 40 |
| Exp19 | Genotype | Gregory | Mp3s3 | 0.78 | 0.61 | 0.60 | 23 | 46 |
| Exp19 | Genotype | Trojan | Mp3s3 | 0.87 | 0.75 | 0.56 | 22 | 44 |
| Exp19 | Density | 75 | Mp3s3 | 0.88 | 0.77 | 0.58 | 28 | 43 |
| Exp19 | Density | 150 | Mp3s3 | 0.75 | 0.56 | 0.61 | 25 | 45 |
| Exp19 | Density | 300 | Mp3s3 | 0.63 | 0.39 | 0.72 | 24 | 42 |
| Exp16 | Management | ILN | Mp3s2 | 0.94 | 0.89 | 0.25 | 24 | 63 |
| Exp16 | Management | RLN | Mp3s2 | 0.95 | 0.90 | 0.32 | 24 | 63 |
| Exp16 | Management | RHN | Mp3s2 | 0.96 | 0.93 | 0.33 | 20 | 63 |
| Exp16 | Management | IHN | Mp3s2 | 0.97 | 0.95 | 0.52 | 31 | 62 |
| Exp19 | Management | RLN | Mp3s3 | 0.64 | 0.41 | 0.52 | 26 | 36 |
| Exp19 | Management | ILN | Mp3s3 | 0.69 | 0.48 | 0.52 | 24 | 36 |
| Exp19 | Management | RHN | Mp3s3 | 0.80 | 0.64 | 0.86 | 30 | 31 |
| Exp19 | Management | IHN | Mp3s3 | 0.90 | 0.82 | 0.61 | 20 | 27 |

Table S3 Paired sample t-test of observed LAI between different levels of four groups (i.e., growing stage characterized by days after sowing (DAS), genotype, plant density and water-nitrogen management) for two field experiments. A tick symbol (“√”) represents the mean of observed LAI between two levels has no difference at 0.05 significant level, and a cross symbol (“×”) represents the mean of observed LAI between two levels are different at 0.05 significant level.

| **Group** | **DAS** |  |  |  |  |  |  |
| --- | --- | --- | --- | --- | --- | --- | --- |
| **Exp16_level** | 18 | 40 | 59 |  |  |  |  |
| 18 | × |  |  |  |  |  |  |
| 40 | √ | × |  |  |  |  |  |
| 40 | √ | √ | × |  |  |  |  |
| **Exp19_level** | 36 | 62 |  |  |  |  |  |
| Exp19_36 | × |  |  |  |  |  |  |
| Exp19_59 | √ | × |  |  |  |  |  |
| **Group** | **Genotype** |  |  |  |  |  |  |
| **Exp16_level** | 7770tin | 7770 | Drysdale | Hartog | Suntop | Spitfire | Gregory |
| 7770tin | × |  |  |  |  |  |  |
| 7770 | √ | × |  |  |  |  |  |
| Drysdale | √ | × | × |  |  |  |  |
| Hartog | √ | √ | × | × |  |  |  |
| Suntop | √ | √ | × | × | × |  |  |
| Spitfire | √ | √ | × | × | × | × |  |
| Gregory | √ | √ | × | × | × | × | × |
| **Exp19_level** | Beaufort | Trojan | Gregory |  |  |  |  |
| Beaufort | × |  |  |  |  |  |  |
| Trojan | × | × |  |  |  |  |  |
| Gregory | × | × | × |  |  |  |  |
| **Group** | **Density** |  |  |  |  |  |  |
| **Exp19_level** | 75 | 150 | 300 |  |  |  |  |
| 75 | × |  |  |  |  |  |  |
| 150 | × | × |  |  |  |  |  |
| 300 | √ | √ | × |  |  |  |  |
| **Group** | **Management** |  |  |  |  |  |  |
| **Exp16_level** | ILN | RLN | RHN | IHN |  |  |  |
| ILN | × |  |  |  |  |  |  |
| RLN | √ | × |  |  |  |  |  |
| RHN | √ | √ | × |  |  |  |  |
| IHN | √ | √ | × | × |  |  |  |
| **Exp19_level** | RLN | ILN | RHN | IHN |  |  |  |
| RLN | × |  |  |  |  |  |  |
| ILN | × | × |  |  |  |  |  |
| RHN | √ | √ | × |  |  |  |  |
| IHN | √ | √ | × | × |  |  |  |
